# The utility of animal models in hiPSC-based studies of autism spectrum disorder: a systematic review

**DOI:** 10.31744/einstein_journal/2026RW1868

**Published:** 2026-09-23

**Authors:** Bruno Yukio Yokota-Moreno, André Luiz Teles e Silva, Isabella de Sousa Nóbrega, Lucas Barbosa Rossetti, Paulo César Maiorka, Andréa Laurato Sertié, Claudia Madalena Cabrera Mori

**Affiliations:** 1 Hospital Israelita Albert Einstein São Paulo SP Brazil Hospital Israelita Albert Einstein, São Paulo, SP, Brazil.; 2 Department of Pathology Faculdade de Medicina Veterinária e Zootecnia Universidade de São Paulo São Paulo SP Brazil Department of Pathology, Faculdade de Medicina Veterinária e Zootecnia, Universidade de São Paulo, São Paulo, SP, Brazil.

**Keywords:** Autism spectrum disorder, Models, animal, Animal use alternatives, Induced pluripotent stem cells, Organoids, Neurodevelopmental disorders, Astrocytes

## Abstract

**Introduction:**

Autism spectrum disorder is a highly prevalent and heritable neurodevelopmental condition. To investigate its pathogenesis, genetic and non-genetic animal models have been developed. However, no animal model can fully capture the human-specific characteristics and genetic complexity of autism spectrum disorder. In this context, human induced pluripotent stem cell (hiPSC) derived models have emerged as powerful tools in autism spectrum disorder research, offering the potential to reduce or replace animal experiments. Despite this potential, many hiPSC-based studies still incorporate animal use.

**Objective:**

To analyze the use of animals in hiPSC-based studies of autism spectrum disorder, with the aim of assessing the necessity of their inclusion and their contribution to our understanding of disease mechanisms.

**Methods:**

Following the PRISMA guidelines, we searched PubMed, Scopus, and Web of Science for articles published between 2009 and 2024 using the search terms “autism”, “ASD”, and “autism spectrum disorder” combined with “iPSC”, “induced pluripotent stem cells and “organoid*”.

**Results:**

A total of 207 studies using hiPSCs to investigate Autism spectrum disorder were identified. Of these, 79 studies (38.16%) also involved animals or animal-derived cells. While nearly half of these studies used autism spectrum disorder animal models to explore neurobiological mechanisms of the disorder, the remaining studies employed animals for purposes not directly related to understanding autism spectrum disorder pathophysiology. These included teratoma formation assays to validate hiPSC pluripotency and the extraction of primary astrocytes for co-culture with hiPSC-derived neurons, an approach that may introduce confounding variables.

**Conclusion:**

Our review highlights the frequent and, in some cases, unnecessary use of animals in hiPSC-based studies of autism spectrum disorder.

## INTRODUCTION

Autism spectrum disorder (ASD) is an early-onset neurodevelopmental condition characterized by impairments in social communication and restricted, repetitive patterns of behavior and interests.^(1)^ In addition to these core symptoms, individuals with ASD often experience co-occurring conditions, including seizures, intellectual disability, gastrointestinal disturbances, and other psychiatric disorders.^(2,3)^ Autism spectrum disorder affects around 2% of the global population and is about four times more prevalent in males than in females.^(4,5)^ The risk of ASD has both genetic and environmental origins, and recent genomic studies have revealed a highly complex genetic architecture, involving both rare high-risk variants and common low-risk variants that contribute to susceptibility.^(6-9)^ This genetic and phenotypic heterogeneity poses significant challenges for researchers aiming to unravel the underlying mechanisms of the disorder.

To address these challenges, various animal models have been developed, including models based on brain lesions, environmental exposures, and genetic modifications.^(10,11)^ While these models have provided valuable insights, their translational relevance has been increasingly questioned due to fundamental interspecies differences in brain anatomy, metabolism, physiology, and gene regulation.^(12,13)^ As an alternative, human induced pluripotent stem cells (hiPSCs) have emerged as powerful tools in ASD research. hiPSCs provide direct access to human-derived biomaterials for disease modeling, drug screening, and the exploration of human-specific developmental pathways.^(14,15)^ Moreover, advances in three-dimensional (3D) culture systems, such as hiPSC-derived neural organoids, have expanded upon traditional two-dimensional (2D) cell culture methods, allowing for more sophisticated modeling of normal and aberrant neurodevelopment.^(16-21)^

Despite the potential of hiPSCs to reduce or replace animal testing in ASD research, hiPSC-based studies continue to incorporate animals in their experimental designs.^(22,23)^

## OBJECTIVE

This systematic review aims to map the use of animals in hiPSC-based studies of autism spectrum disorder and to evaluate the necessity and relevance of their inclusion to improve our understanding of autism spectrum disorder pathogenesis.

## METHODS

### Systematic review

#### Search strategy

This systematic review followed the Preferred Reporting Items for Systematic Reviews and Meta-Analyses (PRISMA) guidelines (https://prisma-statement.org/). A systematic literature search for scientific studies published from January 1, 2009, through October 20, 2024, was conducted in three databases: PubMed, Scopus, and Web of Sciences (Table 1S, Supplementary Material). The following search terms were used: [(“autism” OR “ASD” OR “autism spectrum disorder”) AND (“iPSC” OR “induced pluripotent stem cells” OR “organoid*”)]. The searches were also limited by document type (“Article”) and language (“English”).

#### Eligibility criteria and data extraction

At least two authors independently performed the screening, reviewed full-text articles against the inclusion criteria, and resolved all discrepancies through consensus. Studies were included if they reported experiments involving hiPSCs to investigate the pathophysiology of ASD, were written in English, and were published in peer-reviewed journals. Review articles, commentaries, editorials, and other non-original research formats, as well as in silico studies involving hiPSCs, were excluded. Notably, although article selection was based on the use of hiPSCs in ASD-related research, not all included studies used hiPSCs derived from individuals with ASD. The specific inclusion criteria for the selected articles are detailed in Table 2S, Supplementary Material. Briefly, articles were included if they used: 1) hiPSCs derived from individuals clinically diagnosed with ASD, without or with comorbidities, or Rett syndrome (RTT); 2) genome-edited hiPSCs harboring pathogenic mutations in ASD risk genes; 3) hiPSCs from neurotypical individuals exposed to compounds believed to contribute to ASD pathogenesis.

The following data were extracted from each study: a) the clinical and molecular diagnoses of the individuals who donated primary cells for hiPSCs generation, b) use of hiPSC-derived neural cells in 2D culture or hiPSC-derived 3D neural organoids, c) the use of animals or animal-derived cells, d) the type of animal model employed (if applicable), e) the use of teratoma formation assays, and f) the performance of electrophysiological studies.

## Nomenclature

### Autism spectrum disorder and rett syndrome

In the previous edition of the Diagnostic and Statistical Manual of Mental Disorders (DSM-IV), RTT was classified as an autism spectrum disorder.^(24)^ However, the DSM-5 version published in 2013 no longer includes RTT specifically as an autism spectrum disorder.^(1)^ Despite this reclassification, there are notable phenotypic similarities between RTT and ASD, and many children are diagnosed as autistic before *MECP2* mutations are identified. Therefore, in this review, we also included all studies that met the inclusion criteria and used hiPSCs derived from individuals with RTT.

### Neural organoids

A variety of terms have been used in the literature to describe 3D organoids that mimic the developmental processes and structural organization of the central nervous system, such as SFEBq (serum-free floating culture of embryoid body-like aggregates), neuroloids, and neural spheroids. Additionally, the terms “brain organoids” and “cerebral organoids” have been widely used to refer to unguided organoids that develop heterogeneous regional identities. Moreover, regionalized organoids that resemble specific regions of the nervous system (such as cortical, retinal, hypothalamic, or spinal cord organoids) have been established, further increasing the diversity of the nomenclature. To maintain consistency throughout this review, we adopted the terminology recommended by Pasca et al.,^(21)^ using the broader term “neural organoids” to encompass all forms of brain-related organoids among the articles screened and included in the review.

## RESULTS

### Studies selection

We conducted a systematic literature search for articles published in indexed journals between 2009 and 2024 that used hiPSC-derived models to study ASD. A total of 867 records were screened. The first filtering step resulted in 543 studies, and the second filtering step yielded a final sample of 207 studies (Figure 1) that met the inclusion criteria (Table 2S, Supplementary Material).

**Figure 1:**
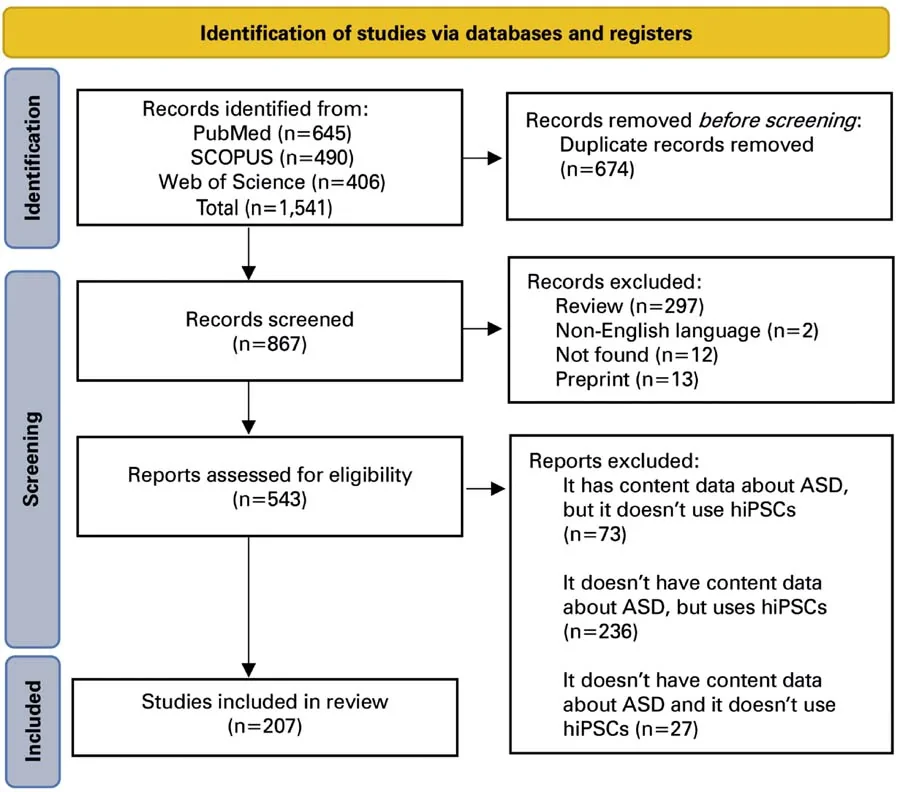
PRISMA 2020 flow diagram summarizing the systematic review process

### Characteristics of the studies selected

We found that 134 studies used hiPSCs derived from individuals diagnosed with ASD, and 11 studies used hiPSCs from individuals with RTT, a condition no longer classified as part of the autism spectrum but sharing notable clinical and neuropathological features with ASD. Additionally, a considerable number of studies (n=55) employed genome-edited hiPSCs carrying pathogenic mutations in genes associated with ASD. Finally, a smaller subset of studies (n=17) used hiPSC-derived cells exposed to specific molecules believed to contribute to ASD pathogenesis, with the aim of replicating environmental risk factors (Figure 2A).

**Figure 2:**
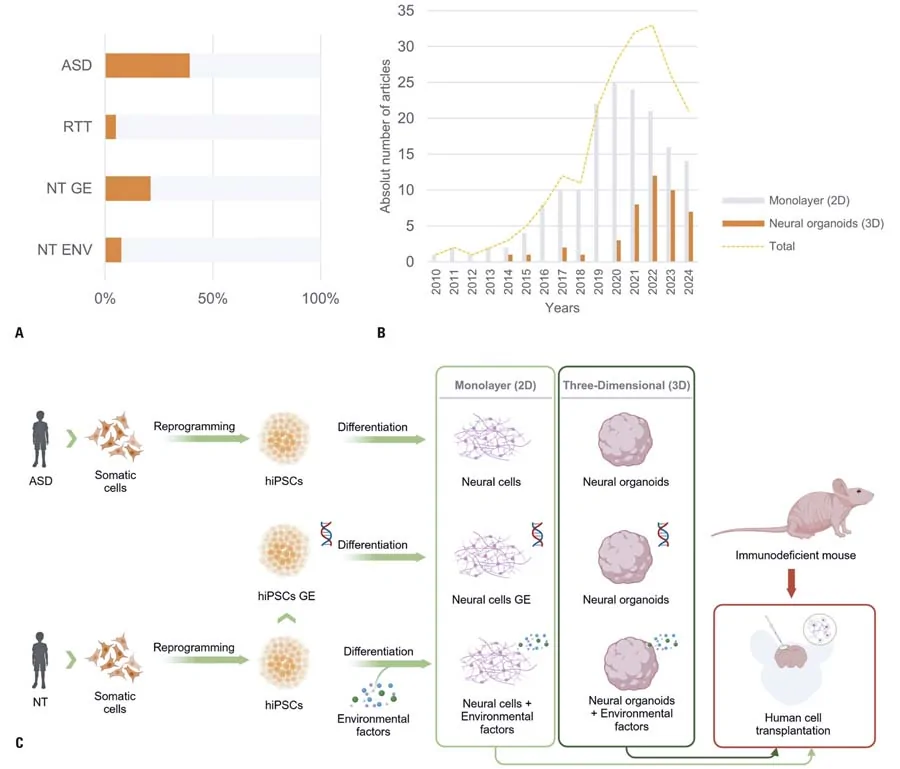
Characteristics of eligible studies and hiPSC-derived models used to model ASD. A) The graph shows the percentage of studies using hiPSCs derived from: individuals with ASD, individuals with RTT, neurotypical (NT) individuals whose hiPSCs underwent genome edited (GE) to introduce mutations in ASD-related genes (NT GE), and NT individuals that were exposed to environmental risk factors (NT ENV). B) The graph shows the number of studies using hiPSC-derived cells in 2D cultures and 3D neural organoid models for ASD research. C) Schematic diagram illustrating the main applications of hiPSCs in ASD research, which include the following: i) differentiation of hiPSCs from individuals with ASD into 2D neural cells or 3D neural organoids; ii) generation of genome-edited hiPSCs with mutations in ASD-related genes and their differentiation into 2D neural cells or 3D neural organoids; iii) differentiation of hiPSCs from NT individuals into 2D neural cells or 3D neural organoids and exposure to environmental risk factors associated with ASD; iv) transplantation of hiPSC-derived 2D neural cells or 3D neural organoids into the brains of immunosuppressed mice

Created using the BioRender tool. Available from: https://www.biorender.com/

While the majority of these studies used hiPSC-derived cells in 2D cultures, recent years have seen a rapid increase in the number of studies using hiPSC-derived 3D neural organoids in ASD research (Figure 2B). Another emerging model involves the transplantation of hiPSC-derived neural cells into the brains of immunosuppressed mice, allowing researchers to study human neural development in an *in vivo* context. However, this approach has been used in only a limited number of recent studies.^(25-29)^Figure 2C provides a schematic diagram illustrating the various hiPSC-based approaches used in ASD research.

We observed that a substantial number of the selected studies employed animals in their experiments. Therefore, we sought to examine how animals were used in these studies to assess the relevance of their inclusion to advance our understanding of ASD pathogenesis. In addition, we sought to evaluate whether alternative, human-based methods could be considered to replace or reduce animal use in this context.

### More than one-third of the studies that used hiPSCs to model autism spectrum disorder also used animals

We observed that, among the 207 studies included in this review, 79 (38.16%) also employed animals or animal-derived cells or tissues in their experimental designs (Figure 3A). Among these studies, most used mice, followed by rats, fish, frogs, pigs, worms, and chickens (Figure 3B). We categorized the use of animals into five main groups: 1) genetically engineered animals carrying mutations in ASD-associated genes; 2) environmentally induced ASD animal models; 3) immunodeficient animals engrafted with hiPSC-derived cells from individuals with ASD; 4) wild-type (WT) animals not used to model ASD but used for purposes other than pathogenesis modeling; and 5) immunodeficient animals not used to model ASD (Figure 3B).

**Figure 3:**
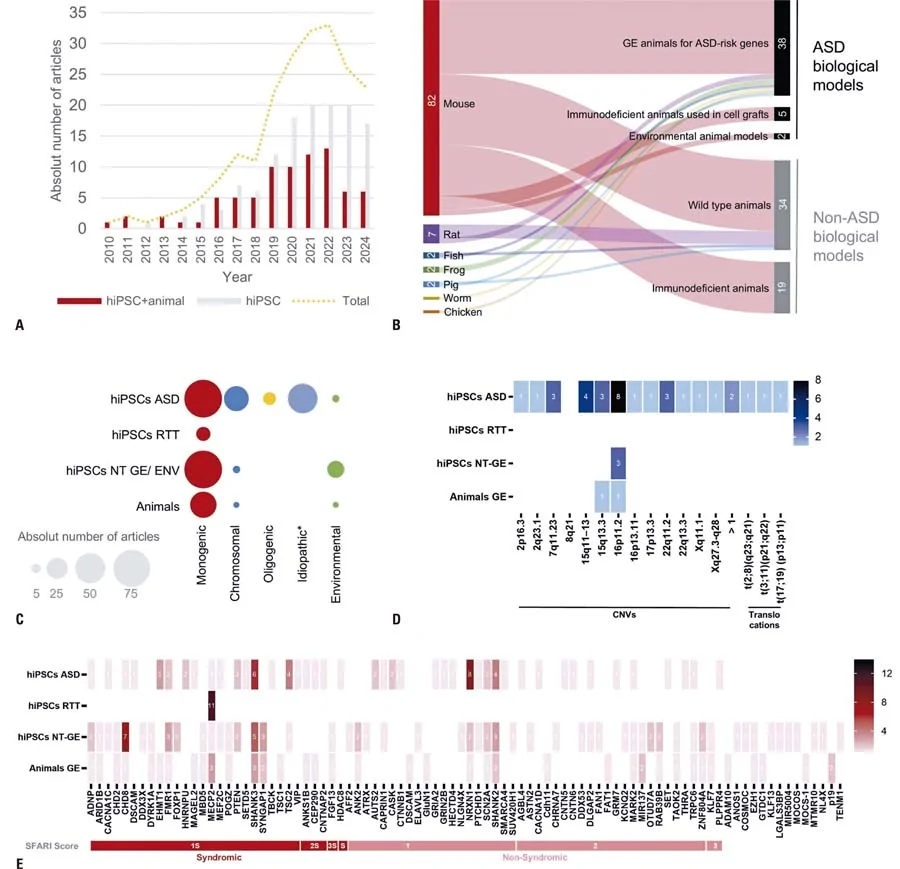
Comparative analysis of studies using hiPSC-derived cells and experimental animals in ASD research. A) The graph shows the number of studies that used only hiPSC-derived models and the number of studies that combined hiPSC- and animal-derived models in ASD research. B) Sankey diagram illustrating the animal species used across the reviewed studies, categorized according to their role in either ASD modeling or other experimental purposes. The number of studies is indicated within the vertical bars. C) Schematic comparison of the number of studies using hiPSC-derived cells* and animal models, categorized according to their genetic backgrounds (monogenic, chromosomal, oligogenic, and idiopathic forms of ASD) or their use as environmental exposure models. D) Schematic diagram showing the number of studies using hiPSC-derived cells* and animal models harboring specific ASD-associated CNVs and chromosomal translocations. E) Schematic diagram showing the number of studies using hiPSC-derived cells* and animal models harboring mutations in single ASD-associated genes, thereby modeling monogenic forms of ASD

*hiPSCs ASD: hiPSC-derived cells from individuals with ASD; hiPSCs RTT: hiPSC-derived cells from individuals with RTT; hiPSCs NT GE: genome-edited hiPSCs from neurotypical individuals carrying mutations in ASD-related genes

Among the 45 studies that used animals to model ASD (groups 1-3), the most commonly used approach was the use of genetically engineered animals carrying ASD-related mutations (n=38 studies) (Figure 3B). These models predominantly involved mice with mutations in single ASD-associated genes, representing monogenic forms of ASD. In contrast, only two studies used mouse models of ASD-associated copy number variants (CNVs) (Figure 3C-E). Notably, none of the studies utilized animal models representing oligogenic or polygenic/idiopathic forms of ASD (Figure 3C), underscoring the significant challenge of creating animal models that accurately replicate the complex genetic landscape of the disorder.

Immunodeficient animals engrafted with hiPSC-derived cells were used in five studies (Figure 3B). These studies involved the transplantation of hiPSC-derived neural or glial cells into the brains of immunodeficient mice, thereby creating human-mouse chimeric brain models. Of these, three studies used cells from individuals with ASD with both known and unknown genetic causes,^(25-27)^ and one study transplanted CRISPR/Cas9-engineered cells with a hemizygous deletion of *SHANK3*, a gene strongly associated with ASD.^(28)^ Another recent study engrafted hiPSC-derived neural organoids from individuals with ASD into the brains of immunodeficient mice.^(29)^

Finally, environmentally induced ASD animal models were the least commonly used, appearing in only two studies (Figure 3B, C). One study exposed pregnant dams to valproic acid, an anticonvulsant and mood stabilizer, to evaluate ASD-like phenotypes in the offspring.^(30)^ The other study administered subcutaneous injections of Zika virus to newborn mice, resulting in behavioral phenotypes consistent with ASD and suggesting a potential association between early developmental exposure to Zika virus and ASD-like phenotypes.^(31)^

Next, we examined the use of animals that were not employed as ASD models (groups 4 and 5, n=53 studies).

### The use of immunodeficient mice to confirm hiPSC pluripotency may be unnecessary

Among the 53 studies that used animals not intended to serve as ASD models, 19 employed immunodeficient mice to conduct teratoma formation assays (TFAs) as a means of validating the hiPSCs pluripotency (Figure 1S, Supplementary Material). The most frequently reported strain was NOD/SCID (n=6 studies), followed by SCID (n=4 studies), and CByJ.Cg-Foxn1nu/J (n=1 study). However, 8 studies did not clearly report which mouse strain was used (Table 3S, Supplementary Material).

When we evaluated the proportion of studies utilizing TFAs relative to the total number of hiPSC-based studies of ASD published each year, we observed a gradual decline in the use of TFAs over time (Figure S1B). In parallel, there was a substantial increase in the use of *in vitro* alternatives, particularly the embryoid body (EB) formation assay, also known as spontaneous differentiation (Figure S1C). In this approach, hiPSCs are cultured in suspension to form 3D aggregates called EBs, which are then plated onto coated surfaces to induce further spontaneous differentiation into cells derived from all three embryonic germ layers.^(32,33)^

We also observed that 31 of the 207 studies (14.97%) were published in the specialized journal Stem Cell Research, which publishes articles describing newly established hiPSC lines. Notably, 24 of these 31 studies utilized the *in vitro* EB formation assay rather than an animal-based TFA to validate pluripotency.

Taken together, these findings suggest that TFAs involving immunodeficient mice may be unnecessary when validated *in vitro* alternative methods are available. Encouragingly, we observed a downward trend in the use of animals for pluripotency validation and a growing preference for non-animal approaches in the characterization of newly established hiPSC lines.

### Use of primary astrocytes derived from wild-type rodents may hinder translational relevance

We identified 34 studies that utilized WT animals (23 used mice, 9 used rats, 1 used a fish, and 1 used pig) with no direct connection to ASD. The majority of these studies employed animals to obtain rodent-derived primary astrocytes (n=26 studies) or neurons (n=3 studies). As neurons co-cultured with astrocytes exhibit more mature firing patterns, we found that 24 of the 26 studies using rodent astrocytes co-cultured them with hiPSC-derived neurons from individuals with ASD for electrophysiological recordings (Figure 4).

**Figure 4:**
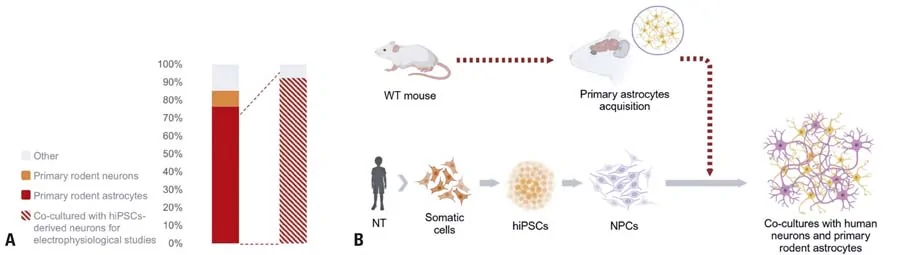
Use of rodent primary astrocytes co-cultured with hiPSC-derived neurons for electrophysiological recordings. A) The graph on the left shows the percentage of studies using primary astrocytes and neurons isolated from WT rodents. The graph on the right illustrates the percentage of studies employing rodent primary astrocytes in co-culture with hiPSC-derived neurons for electrophysiological analyses. B) Schematic diagram illustrating the main steps involved in isolating primary astrocytes from WT rodents and co-culturing them with hiPSC-derived neural progenitor cells (NPCs) to support neuronal maturation and promote more mature firing patterns

Created using the BioRender tool. Available from: https://www.biorender.com/

Notably, we observed a lack of detailed information regarding the strain or genetic background, sex, age, and number of rodents used in these studies (Table 1). Therefore, in addition to the well-documented physiological differences between rodent and human astrocytes, the use of primary rodent astrocytes in co-culture experiments with hiPSC-derived neurons may compromise reproducibility and complicate the interpretation of the pathophysiology of ASD.

**Table 1:** Information on the rodents used to isolate primary astrocytes. An x indicates that the information was not provided or was unclear

Animal	Background	Sex	Age	Nº	Region of the brain	Reference
Mouse	x	x	x	x	Cerebral cortex	Brick et al., 2014^(34)^
Mouse	x	x	PD 3-5	x	Cerebral cortex	Tang et al., 2016^(35)^
Rat	x	x	x	x	Hippocampus	Marchetto et al., 2017^(36)^
Mouse	CD-1	x	PD 1	x	x	Deneault et al., 2018^(37)^
Mouse	x	x	PD 5-7	x	Cerebellum	Sundberg et al., 2018^(38)^
Rat	x	x	x	x	x	Carbonell et al., 2019^(39)^
Mouse	CD-1	x	PD 1	x	x	Deneault et al., 2019^(40)^
Rat	x	x	PD 0-3	x	x	Huang et al., 2019^(41)^
Mouse	x	x	x	x	x	Sengar et al., 2019^(42)^
Mouse	ICR	x	PD 0	x	Cerebral cortex	Song et al., 2019^(43)^
Mouse	x	x	x	x	x	Zaslavsky et al., 2019^(44)^
Rat	X	x	x	x	x	Llamosas et al., 2020^(45)^
Mouse	CD-1	x	x	x	x	Ross et al., 2020^(46)^
Rat	x	x	x	x	Cerebral cortex	Meganathan et al., 2021^(47)^
Rat	x	x	x	x	x	Kozlova et al., 2022^(48)^
Mouse	x	x	PD 1	x	x	Mok et al., 2022^(49)^
Mouse	x	x	x	x	Cerebral cortex	Murtaza et al., 2022^(50)^
Mouse	x	x	x	x	x	Nehme et al., 2022^(51)^
Rat	x	x	x	x	x	Teunissen et al., 2023^(52)^
Mouse	CD-1 mice	x	E16	x	Cerebral cortex	Unda et al., 2023^(53)^
Mouse	CD-1 mice	x	PD 1	x	Cerebral cortex	Brown et al., 2024^(54)^
Mouse	x	x	PD 0-3	x	x	Lai et al., 2024^(55)^
Mouse	C57BL/6	x	PD 1-2	x	Cerebral cortex	Trudler et al., 2025^(56)^

PD: postnatal day; E: embryonic day.

## DISCUSSION

In this systematic review, we found that 79 studies (38.16%) using hiPSCs in ASD research also employed animals or animal-derived cells. While nearly half of these studies used ASD animal models to investigate the neurobiological mechanisms of the disorder, the remaining studies involved animals in experiments not directly aimed at understanding the pathophysiology of ASD or in ways that could introduce confounding factors.

Although animal models have advanced our knowledge of ASD, accurately replicating the genetic complexity of the disorder in animals remains challenging.^(57)^ We found that most studies utilized genetically modified animals to model monogenic forms of ASD, with limited representation of oligogenic, chromosomal, or polygenic forms. In this context, hiPSC-derived cells transplanted into the mouse brain represent a promising strategy to bridge the translational gap between preclinical animal models and human studies.^(58,59)^ By using hiPSCs derived from individuals with ASD with diverse genetic backgrounds, researchers can create more physiologically relevant models that better capture the genetic heterogeneity of human ASD.^(25-27,29)^ Compared with genetically engineered animals, humanized transplantation models may also reduce animal use in experiments by reducing the large numbers typically required for the breeding and maintenance of genetically modified lines. It is estimated that, just in the European Union alone, around 14 million animals are culled annually due to an unsuitable genotype, sex, or age.^(60)^ Notably, we found that some studies reported mortality rates exceeding 90% in genetically modified mice within the first two months of life.^(61)^ In addition, ASD is about four times more prevalent in males than in females, contributing to a bias toward the use of male rodents in research. Conversely, RTT, which primarily affects females, may lead to a greater use of female animals in RTT research. ^(62,63)^ Therefore, the use of humanized transplantation models highlights the potential of hiPSCs to reduce and refine animal use.

We also found frequent use of immunodeficient mice in TFAs to validate hiPSC pluripotency, a practice that raises significant animal welfare concerns. Although TFAs were once considered the gold standard, their use has declined over time alongside an increase in *in vitro* spontaneous differentiation approaches, such as EB formation assays, which may provide a comparable assessment of pluripotency. This transition illustrates how accessible alternative technologies can effectively reduce animal use in research.

It is also notable that a substantial number of studies used WT animals to isolate primary astrocytes for co-culture with hiPSC-derived neurons during electrophysiological recordings. Accumulating evidence demonstrates that astrocytes from individuals with ASD exhibit altered properties and contribute substantially to neuronal development, physiology, and behavior.^(27,64-67)^ Therefore, reliance on animal-derived astrocytes risks generating inaccurate or misleading findings. Compounding this concern, many studies failed to report critical animal details such as strain, sex, age, and the number of animals used, which may undermine reproducibility and contribute to the ongoing reproducibility crisis in biomedical research.^(68,69)^

The advent of neural organoid technology presents an opportunity to address some of the drawbacks of animal experimentation. Neural organoids contain diverse brain cell types, including neurons, astrocytes, and neural progenitors,^(16,70-72)^ as well as cell types that are abundant in humans but nearly absent in rodents, such as outer radial glial cells.^(23)^ While mouse brain maturation occurs over approximately 1.5 months, neural organoids can be cultured for years, more faithfully recapitulating the timeline of human brain development.^(73,74)^ Studies have demonstrated that six-month-old neural organoids generate intricate oscillatory electrical activity reminiscent of human neonatal electroencephalography patterns, suggesting their potential to model aspects of human brain function that may not be fully captured by 2D cultures or animal models.^(75)^ Although not yet applied in ASD research, organ-on-a-chip technologies^(76)^ hold further promise for reducing animal use by integrating hiPSC-derived neural cells within biomimetic environments that replicate key features of the human brain.

Transitioning from 2D cultures to 3D organoids and organ-on-a-chip platforms, alongside the refinement of these models for reproducibility and complexity, may reduce animal use and improve the quality of ASD research. However, many protocols require adaptation to achieve truly animal-free human models. For instance, Matrigel, a widely used matrix for supporting organoid development,^(16)^ is derived from the extracellular matrix of mouse tumors.^(77)^ Similarly, commonly used culture medium supplements, such as fetal bovine serum and porcine-based heparin, introduce additional animal-derived components that could be replaced with chemically defined alternatives.^(78)^

Finally, although this review focuses on ASD, it is possible that similar patterns of unnecessary animal use occur in research on other neurodevelopmental disorders. Our findings provide guidance for identifying and reducing potentially unnecessary animal use in biomedical research.

## CONCLUSION

In conclusion, while animal models have played a fundamental role in autism spectrum disorder research, the emergence of hiPSC-based models offers new opportunities for more human-relevant and ethically responsible approaches. Systematically identifying and addressing unnecessary or inappropriate uses of animals not only improves the scientific rigor and translational value of autism spectrum disorder studies but also supports the growing imperative to replace, reduce, and refine animal experimentation in biomedical research.

## SUPPLEMENTARY MATERIAL

Supplementary Material

## References

[1] Association AP (2013). Diagnostic and Statistical Manual of Mental Disorders (DSM-V).

[2] Bougeard C, Picarel-Blanchot F, Schmid R, Campbell R, Buitelaar J (2021). Prevalence of Autism Spectrum Disorder and Co-morbidities in Children and Adolescents: A Systematic Literature Review. Front Psychiatry.

[3] Khachadourian V, Mahjani B, Sandin S, Kolevzon A, Buxbaum JD, Reichenberg A (2023). Comorbidities in autism spectrum disorder and their etiologies. Transl Psychiatry.

[4] Zeidan J, Fombonne E, Scorah J, Ibrahim A, Durkin MS, Saxena S (2022). Global prevalence of autism: A systematic review update. Autism Res.

[5] Maenner MJ, Warren Z, Williams AR, Amoakohene E, Bakian AV, Bilder DA (2023). Prevalence and Characteristics of Autism Spectrum Disorder Among Children Aged 8 Years - Autism and Developmental Disabilities Monitoring Network, 11 Sites, United States, 2020. MMWR Surveill Summ.

[6] Grove J, Ripke S, Als TD, Mattheisen M, Walters RK, Won H, Pallesen J, Agerbo E, Andreassen OA, Anney R, Awashti S, Belliveau R, Bettella F, Buxbaum JD, Bybjerg-Grauholm J, Bækvad-Hansen M, Cerrato F, Chambert K, Christensen JH, Churchhouse C, Dellenvall K, Demontis D, De Rubeis S, Devlin B, Djurovic S, Dumont AL, Goldstein JI, Hansen CS, Hauberg ME, Hollegaard MV, Hope S, Howrigan DP, Huang H, Hultman CM, Klei L, Maller J, Martin J, Martin AR, Moran JL, Nyegaard M, Nærland T, Palmer DS, Palotie A, Pedersen CB, Pedersen MG, dPoterba T, Poulsen JB, Pourcain BS, Qvist P, Rehnström K, Reichenberg A, Reichert J, Robinson EB, Roeder K, Roussos P, Saemundsen E, Sandin S, Satterstrom FK, Davey Smith G, Stefansson H, Steinberg S, Stevens CR, Sullivan PF, Turley P, Walters GB, Xu X, Stefansson K, Geschwind DH, Nordentoft M, Hougaard DM, Werge T, Mors O, Mortensen PB, Neale BM, Daly MJ, Børglum AD, Autism Spectrum Disorder Working Group of the Psychiatric Genomics Consortium, BUPGEN, Major Depressive Disorder Working Group of the Psychiatric Genomics Consortium, 23andMe Research Team (2019). Identification of common genetic risk variants for autism spectrum disorder. Nat Genet.

[7] Satterstrom FK, Kosmicki JA, Wang J, Breen MS, De Rubeis S, An JY, Peng M, Collins R, Grove J, Klei L, Stevens C, Reichert J, Mulhern MS, Artomov M, Gerges S, Sheppard B, Xu X, Bhaduri A, Norman U, Brand H, Schwartz G, Nguyen R, Guerrero EE, Dias C, Betancur C, Cook EH, Gallagher L, Gill M, Sutcliffe JS, Thurm A, Zwick ME, Børglum AD, State MW, Cicek AE, Talkowski ME, Cutler DJ, Devlin B, Sanders SJ, Roeder K, Daly MJ, Buxbaum JD, Autism Sequencing Consortium, iPSYCH-Broad Consortium (2020). Large-Scale Exome Sequencing Study Implicates Both Developmental and Functional Changes in the Neurobiology of Autism. Cell.

[8] Fu JM, Satterstrom FK, Peng M, Brand H, Collins RL, Dong S, Wamsley B, Klei L, Wang L, Hao SP, Stevens CR, Cusick C, Babadi M, Banks E, Collins B, Dodge S, Gabriel SB, Gauthier L, Lee SK, Liang L, Ljungdahl A, Mahjani B, Sloofman L, Smirnov AN, Barbosa M, Betancur C, Brusco A, Chung BHY, Cook EH, Cuccaro ML, Domenici E, Ferrero GB, Gargus JJ, Herman GE, Hertz-Picciotto I, Maciel P, Manoach DS, Passos-Bueno MR, Persico AM, Renieri A, Sutcliffe JS, Tassone F, Trabetti E, Campos G, Cardaropoli S, Carli D, Chan MCY, Fallerini C, Giorgio E, Girardi AC, Hansen-Kiss E, Lee SL, Lintas C, Ludena Y, Nguyen R, Pavinato L, Pericak-Vance M, Pessah IN, Schmidt RJ, Smith M, Costa CIS, Trajkova S, Wang JYT, Yu MHC, Cutler DJ, De Rubeis S, Buxbaum JD, Daly MJ, Devlin B, Roeder K, Sanders SJ, Talkowski ME, Autism Sequencing Consortium (ASC), Broad Institute Center for Common Disease Genomics (Broad-CCDG), iPSYCH-BROAD Consortium (2022). Rare coding variation provides insight into the genetic architecture and phenotypic context of autism. Nat Genet.

[9] Zhou X, Feliciano P, Shu C, Wang T, Astrovskaya I, Hall JB, Obiajulu JU, Wright JR, Murali SC, Xu SX, Brueggeman L, Thomas TR, Marchenko O, Fleisch C, Barns SD, Snyder LG, Han B, Chang TS, Turner TN, Harvey WT, Nishida A, O'Roak BJ, Geschwind DH, Michaelson JJ, Volfovsky N, Eichler EE, Shen Y, Chung WK, SPARK Consortium (2022). Integrating de novo and inherited variants in 42,607 autism cases identifies mutations in new moderate-risk genes. Nat Genet.

[10] Kim KC, Gonzales EL, Lázaro MT, Choi CS, Bahn GH, Yoo HJ (2016). Clinical and Neurobiological Relevance of Current Animal Models of Autism Spectrum Disorders. Biomol Ther.

[11] Varghese M, Keshav N, Jacot-Descombes S, Warda T, Wicinski B, Dickstein DL (2017). Autism spectrum disorder: neuropathology and animal models. Acta Neuropathol.

[12] Silverman JL, Thurm A, Ethridge SB, Soller MM, Petkova SP, Abel T (2022). Reconsidering animal models used to study autism spectrum disorder: current state and optimizing future. Genes Brain Behav.

[13] Pound P, Ritskes-Hoitinga M (2018). Is it possible to overcome issues of external validity in preclinical animal research? Why most animal models are bound to fail. J Transl Med.

[14] Takahashi K, Yamanaka S (2006). Induction of pluripotent stem cells from mouse embryonic and adult fibroblast cultures by defined factors. Cell.

[15] Guerreiro S, Maciel P (2023). Transition from Animal-Based to Human Induced Pluripotent Stem Cells (iPSCs)-Based Models of Neurodevelopmental Disorders: opportunities and Challenges. Cells.

[16] Lancaster MA, Renner M, Martin CA, Wenzel D, Bicknell LS, Hurles ME (2013). Cerebral organoids model human brain development and microcephaly. Nature.

[17] Velasco S, Kedaigle AJ, Simmons SK, Nash A, Rocha M, Quadrato G (2019). Individual brain organoids reproducibly form cell diversity of the human cerebral cortex. Nature.

[18] Kanton S, Boyle MJ, He Z, Santel M, Weigert A, Sanchís-Calleja F (2019). Organoid single-cell genomic atlas uncovers human-specific features of brain development. Nature.

[19] Dixon TA, Muotri AR (2023). Advancing preclinical models of psychiatric disorders with human brain organoid cultures. Mol Psychiatry.

[20] Bock C, Boutros M, Camp JG, Clarke L, Clevers H, Knoblich JA, Liberali P, Regev A, Rios AC, Stegle O, Stunnenberg HG, Teichmann SA, Treutlein B, Vries RG, Human Cell, Atlas 'Biological, Network' Organoids (2021). The Organoid Cell Atlas. Nat Biotechnol.

[21] Pa?ca SP, Arlotta P, Bateup HS, Camp JG, Cappello S, Gage FH (2022). A nomenclature consensus for nervous system organoids and assembloids. Nature.

[22] Lim CS, Kim MJ, Choi JE, Islam MA, Lee YK, Xiong Y (2021). Dysfunction of NMDA receptors in neuronal models of an autism spectrum disorder patient with a DSCAM mutation and in Dscam-knockout mice. Mol Psychiatry.

[23] Zhang W, Ma L, Yang M, Shao Q, Xu J, Lu Z (2020). Cerebral organoid and mouse models reveal a RAB39b-PI3K-mTOR pathway-dependent dysregulation of cortical development leading to macrocephaly/autism phenotypes. Genes Dev.

[24] Association AP (1994). Diagnostic and Statistical Manual of Mental Disorders.

[25] Vitrac A, Pons S, Balkota M, Lemière N, Raïs C, Bourgeois JP (2020). A chimeric mouse model to study human iPSC-derived neurons: the case of a truncating SHANK3 mutation. Sci Rep.

[26] Li J, Brickler T, Banuelos A, Marjon K, Shcherbina A, Banerjee S (2021). Overexpression of CD47 is associated with brain overgrowth and 16p11.2 deletion syndrome. Proc Natl Acad Sci USA.

[27] Allen M, Huang BS, Notaras MJ, Lodhi A, Barrio-Alonso E, Lituma PJ (2022). Astrocytes derived from ASD individuals alter behavior and destabilize neuronal activity through aberrant Ca ^2+^ signaling. Mol Psychiatry.

[28] Chiola S, Napan KL, Wang Y, Lazarenko RM, Armstrong CJ, Cui J (2021). Defective AMPA-mediated synaptic transmission and morphology in human neurons with hemizygous SHANK3 deletion engrafted in mouse prefrontal cortex. Mol Psychiatry.

[29] Schafer ST, Mansour AA, Schlachetzki JC, Pena M, Ghassemzadeh S, Mitchell L (2023). An in vivo neuroimmune organoid model to study human microglia phenotypes. Cell.

[30] Rhinn M, Zapata-Bodalo I, Klein A, Plassat JL, Knauer-Meyer T, Keyes WM (2022). Aberrant induction of p19Arf-mediated cellular senescence contributes to neurodevelopmental defects. PLoS Biol.

[31] Ohki CM, Benazzato C, van der Linden V, França JV, Toledo CM, Machado RR (2024). Zika virus infection impairs synaptogenesis, induces neuroinflammation, and could be an environmental risk factor for autism spectrum disorder outcome. Biochim Biophys Acta Mol Basis Dis.

[32] Kamand M, Ilieva M, Forsberg SL, Thomassen M, Fex Svenningsen Å, Holst B (2020). Generation of human induced pluripotent stem cells (SDUKIi002-A) from a 22-year-old male diagnosed with autism spectrum disorder. Stem Cell Res.

[33] Yang L, Lu S, Yang Z, Yao J, Zhou P, Zhao M (2023). Generation of an induced pluripotent stem cell line (IPCASi001-A) from an autism spectrum disorder individual without intellectual disability. Stem Cell Res.

[34] Brick DJ, Nethercott HE, Montesano S, Banuelos MG, Stover AE, Schutte SS (2014). The Autism Spectrum Disorders Stem Cell Resource at Children's Hospital of Orange County: Implications for Disease Modeling and Drug Discovery. Stem Cells Transl Med.

[35] Tang X, Kim J, Zhou L, Wengert E, Zhang L, Wu Z (2016). KCC2 rescues functional deficits in human neurons derived from patients with Rett syndrome. Proc Natl Acad Sci USA.

[36] Marchetto MC, Belinson H, Tian Y, Freitas BC, Fu C, Vadodaria K (2017). Altered proliferation and networks in neural cells derived from idiopathic autistic individuals. Mol Psychiatry.

[37] Deneault E, White SH, Rodrigues DC, Ross PJ, Faheem M, Zaslavsky K (2018). Complete Disruption of Autism-Susceptibility Genes by Gene Editing Predominantly Reduces Functional Connectivity of Isogenic Human Neurons. Stem Cell Reports.

[38] Sundberg M, Tochitsky I, Buchholz DE, Winden K, Kujala V, Kapur K (2018). Purkinje cells derived from TSC patients display hypoexcitability and synaptic deficits associated with reduced FMRP levels and reversed by rapamycin. Mol Psychiatry.

[39] Carbonell AU, Cho CH, Tindi JO, Counts PA, Bates JC, Erdjument-Bromage H (2019). Haploinsufficiency in the ANKS1B gene encoding AIDA-1 leads to a neurodevelopmental syndrome. Nat Commun.

[40] Deneault E, Faheem M, White SH, Rodrigues DC, Sun S, Wei W (2019). CNTN5-/+or EHMT2-/+human iPSC-derived neurons from individuals with autism develop hyperactive neuronal networks. eLife.

[41] Huang G, Chen S, Chen X, Zheng J, Xu Z, Doostparast Torshizi A (2019). Uncovering the Functional Link Between SHANK3 Deletions and Deficiency in Neurodevelopment Using iPSC-Derived Human Neurons. Front Neuroanat.

[42] Sengar AS, Li H, Zhang W, Leung C, Ramani AK, Saw NM (2019). Control of Long-Term Synaptic Potentiation and Learning by Alternative Splicing of the NMDA Receptor Subunit GluN1. Cell Rep.

[43] Song J, Yang X, Zhou Y, Chen L, Zhang X, Liu Z (2019). Dysregulation of neuron differentiation in an autistic savant with exceptional memory. Mol Brain.

[44] Zaslavsky K, Zhang WB, McCready FP, Rodrigues DC, Deneault E, Loo C (2019). SHANK2 mutations associated with autism spectrum disorder cause hyperconnectivity of human neurons. Nat Neurosci.

[45] Llamosas N, Arora V, Vij R, Kilinc M, Bijoch L, Rojas C (2020). SYNGAP1 Controls the Maturation of Dendrites, Synaptic Function, and Network Activity in Developing Human Neurons. J Neurosci.

[46] Ross PJ, Zhang WB, Mok RS, Zaslavsky K, Deneault E, D'Abate L (2020). Synaptic Dysfunction in Human Neurons With Autism-Associated Deletions in PTCHD1-AS. Biol Psychiatry.

[47] Meganathan K, Prakasam R, Baldridge D, Gontarz P, Zhang B, Urano F (2021). Altered neuronal physiology, development, and function associated with a common chromosome 15 duplication involving CHRNA7. BMC Biol.

[48] Kozlova A, Zhang S, Kotlar AV, Jamison B, Zhang H, Shi S (2022). Loss of function of OTUD7A in the schizophrenia- associated 15q13.3 deletion impairs synapse development and function in human neurons. Am J Hum Genet.

[49] Mok RS, Zhang W, Sheikh TI, Pradeepan K, Fernandes IR, DeJong LC (2022). Wide spectrum of neuronal and network phenotypes in human stem cell-derived excitatory neurons with Rett syndrome-associated MECP2 mutations. Transl Psychiatry.

[50] Murtaza N, Cheng AA, Brown CO, Meka DP, Hong S, Uy JA (2022). Neuron-specific protein network mapping of autism risk genes identifies shared biological mechanisms and disease-relevant pathologies. Cell Rep.

[51] Nehme R, Pietiläinen O, Artomov M, Tegtmeyer M, Valakh V, Lehtonen L (2022). The 22q11.2 region regulates presynaptic gene-products linked to schizophrenia. Nat Commun.

[52] Teunissen MW, Lewerissa E, van Hugte EJ, Wang S, Ockeloen CW, Koolen DA (2023). ANK2 loss-of-function variants are associated with epilepsy, and lead to impaired axon initial segment plasticity and hyperactive network activity in hiPSC-derived neuronal networks. Hum Mol Genet.

[53] Unda BK, Chalil L, Yoon S, Kilpatrick S, Irwin C, Xing S (2023). Impaired OTUD7A-dependent Ankyrin regulation mediates neuronal dysfunction in mouse and human models of the 15q13.3 microdeletion syndrome. Mol Psychiatry.

[54] Brown CO, Uy JA, Murtaza N, Rosa E, Alfonso A, Dave BM (2024). Disruption of the autism-associated gene SCN2A alters synaptic development and neuronal signaling in patient iPSC-glutamatergic neurons. Front Cell Neurosci.

[55] Lai W, Zhao Y, Chen Y, Dai Z, Chen R, Niu Y (2024). Autism patient-derived SHANK2B Y29X mutation affects the development of ALDH1A1 negative dopamine neuron. Mol Psychiatry.

[56] Trudler D, Ghatak S, Bula M, Parker J, Talantova M, Luevanos M (2025). Dysregulation of miRNA expression and excitation in MEF2C autism patient hiPSC-neurons and cerebral organoids. Mol Psychiatry.

[57] Watanabe N, Kitada K, Santostefano KE, Yokoyama A, Waldrop SM, Heldermon CD (2020). Generation of Induced Pluripotent Stem Cells from a Female Patient with a Xq27.3-q28 Deletion to Establish Disease Models and Identify Therapies. Cell Reprogram.

[58] Mansour AA, Gonçalves JT, Bloyd CW, Li H, Fernandes S, Quang D (2018). An in vivo model of functional and vascularized human brain organoids. Nat Biotechnol.

[59] Dong X, Xu SB, Chen X, Tao M, Tang XY, Fang KH (2021). Human cerebral organoids establish subcortical projections in the mouse brain after transplantation. Mol Psychiatry.

[60] Lewejohann L, Grune B, Schönfelder G, Bert B (2020). Cut back on surplus laboratory animals. Nature.

[61] Tian H, Qiao S, Zhao Y, Jin X, Wang C, Wang R (2022). Krüppel-like Transcription Factor 7 Is a Causal Gene in Autism Development. Int J Mol Sci.

[62] Ip JP, Mellios N, Sur M (2018). Rett syndrome: insights into genetic, molecular and circuit mechanisms. Nat Rev Neurosci.

[63] Petriti U, Dudman DC, Scosyrev E, Lopez-Leon S (2023). Global prevalence of Rett syndrome: systematic review and meta-analysis. Syst Rev.

[64] Russo FB, Freitas BC, Pignatari GC, Fernandes IR, Sebat J, Muotri AR (2018). Modeling the Interplay Between Neurons and Astrocytes in Autism Using Human Induced Pluripotent Stem Cells. Biol Psychiatry.

[65] Vakilzadeh G, Falcone C, Dufour B, Hong T, Noctor SC, Martínez-Cerdeño V (2022). Decreased number and increased activation state of astrocytes in gray and white matter of the prefrontal cortex in autism. Cereb Cortex.

[66] Nassir N, Bankapur A, Samara B, Ali A, Ahmed A, Inuwa IM (2021). Single-cell transcriptome identifies molecular subtype of autism spectrum disorder impacted by de novo loss-of-function variants regulating glial cells. Hum Genomics.

[67] Velmeshev D, Schirmer L, Jung D, Haeussler M, Perez Y, Mayer S (2019). Single-cell genomics identifies cell type-specific molecular changes in autism. Science.

[68] Baker M (2016). 1,500 scientists lift the lid on reproducibility. Nature.

[69] Frommlet F (2020). Improving reproducibility in animal research. Sci Rep.

[70] Pasca AM, Sloan SA, Clarke LE, Tian Y, Makinson CD, Huber N (2015). Functional cortical neurons and astrocytes from human pluripotent stem cells in 3D culture. Nat Methods.

[71] Sloan SA, Andersen J, Pa?ca AM, Birey F, Pa?ca SP (2018). Generation and assembly of human brain region-specific three-dimensional cultures. Nat Protoc.

[72] Jourdon A, Wu F, Mariani J, Capauto D, Norton S, Tomasini L (2023). Modeling idiopathic autism in forebrain organoids reveals an imbalance of excitatory cortical neuron subtypes during early neurogenesis. Nat Neurosci.

[73] Lindhout FW, Krienen FM, Pollard KS, Lancaster MA (2024). A molecular and cellular perspective on human brain evolution and tempo. Nature.

[74] Gordon A, Yoon SJ, Tran SS, Makinson CD, Park JY, Andersen J (2021). Long-term maturation of human cortical organoids matches key early postnatal transitions. Nat Neurosci.

[75] Trujillo CA, Gao R, Negraes PD, Gu J, Buchanan J, Preissl S (2019). Complex Oscillatory Waves Emerging from Cortical Organoids Model Early Human Brain Network Development. Cell Stem Cell.

[76] Servais B, Mahmoudi N, Gautam V, Tong W, Ibbotson MR, Nisbet DR (2024). Engineering brain-on-a-chip platforms. Nat Rev Bioeng.

[77] Kozlowski MT, Crook CJ, Ku HT (2021). Towards organoid culture without Matrigel. Commun Biol.

[78] Ormel PR, Vieira de Sá R, van Bodegraven EJ, Karst H, Harschnitz O, Sneeboer MA (2018t). Microglia innately develop within cerebral organoids. Nat Commun.

