# Supplementary material for: The utility of animal models in hiPSC-based studies of autism spectrum disorder: a systematic review
Source: Einstein (Sao Paulo). 2026 Sep 23;24:eRW1868. doi: 10.31744/einstein_journal/2026RW1868 (PMC13682788; doi:10.31744/einstein_journal/2026RW1868)
Supplement: Supplementary file 1 [file 2317-6385-eins-24-eRW1868-suppl01.pdf]

SUPPLEMENTARY MATERIAL

The utility of animal models in hiPSC-based studies of autism spectrum disorder: a systematic review

Bruno Yukio Yokota-Moreno, André Luiz Teles e Silva, Isabella de Sousa Nóbrega, Lucas Barbosa Rossetti, Paulo César Maiorka, Andréa Laurato Sertié*, Claudia Madalena Cabrera Mori*

* Jointly supervised and contributed equally to this work.

DOI: 10.31744/einstein_journal/2026RW1868

Table 1S. Database search strategy

Database	Search Strategy	Filters	Number of articles found
PubMed	("autism" OR "ASD" OR "autism spectrum disorder") AND ("iPSC" OR "induced pluripotent stem cells" OR "organoid*")	-	645
SCOPUS	TITLE-ABS-KEY ("Autism" OR "Autism Spectrum Disorder" OR "ASD") AND TITLE-ABS-KEY ("iPSC" OR "induced pluripotent stem cells" OR "organoid*") AND (LIMIT-TO (DOCTYPE , «ar")) AND (LIMIT-TO (LANGUAGE , «English"))	-	490
Web of Sciences	ALL=("autism" OR "autism spectrum disorder" OR "ASD") AND ALL=("iPSC" OR "induced pluripotent stem cell" OR "organoid*")	Limited by: "Article"; Language: "English"	406

Table 2S. Inclusion criteria for each selected article

Year	Title	Journal	Iclusion criteria	ASD	RTT	CTRL GM	ASD ENV	CTRL ENV	2D/3D	Electrophysiological studies	Use animals
2010	A model for neural development and treatment of Rett syndrome using human induced pluripotent stem cells	Cell	Use iPSCs from Ret Syndrome (RTT) patients - a disorder strongly associated with ASD		x				2D	Yes	Yes
2011	Isogenic pairs of wild type and mutant induced pluripotent stem cell (iPSC) lines from Rett syndrome patients as *in vitro* disease model	PLoS One	Use iPSCs from Ret Syndrome (RTT) patients - a disorder strongly associated with ASD		x				2D	No	Yes
2011	Isolation of MECP2-null Rett Syndrome patient hiPS cells and isogenic controls through X-chromosome inactivation	Hum Mol Genet	Use iPSCs from Ret Syndrome (RTT) patients - a disorder strongly associated with ASD		x				2D	No	Yes
2012	Derivation of autism spectrum disorder-specific induced pluripotent stem cells from peripheral blood mononuclear cells	Neurosci Lett	Use iPSCs from ASD patients	x					2D	No	No
2013	MeCP2 regulates the synaptic expression of a Dysbindin-BLOC-1 network component in mouse brain and human induced pluripotent stem cell-derived neurons	PLoS One	Use iPSCs from Ret Syndrome (RTT) patients - a disorder strongly associated with ASD		x				2D	No	Yes
2013	Functional impacts of NRXN1 knockdown on neurodevelopment in stem cell models	PLoS One	Use NRXN1-knockdown-iPSCs - a condition strongly associated with ASD			x			2D	No	Yes
2014	The Autism Spectrum Disorders Stem Cell Resource at Children's Hospital of Orange County: Implications for Disease Modeling and Drug Discovery	Stem Cells Transl Med	Use iPSCs from ASD and other related conditions	x					2D	Yes	Yes

continue...

...Continuation

Table 2S. Inclusion criteria for each selected article

Year	Title	Journal	Iclusion criteria	ASD	RTT	CTRL GM	ASD ENV	CTRL ENV	2D/3D	Electrophysiological studies	Use animals
2014	iPSC-derived forebrain neurons from FXS individuals show defects in initial neurite outgrowth	Stem Cells Dev	Use iPSCs from individuals with ASD and Fragile X Syndrome	x					2D	No	No
2014	Heat shock alters the expression of schizophrenia and autism candidate genes in an induced pluripotent stem cell model of the human telencephalon	PLoS One	Use a Heat shock-iPSC-derived model to understand the potential effect of fever on the developing human brain and found some ASD genes differentially expressed					x	Neural organoid	No	No
2015	ZNF804A Transcriptional Networks in Differentiating Neurons Derived from Induced Pluripotent Stem Cells of Human Origin	PLoS One	Use a ZNF804A knockdown-iPSCs - ZNF804A is a gene associated with ASD			x			2D	No	No
2015	Modeling non-syndromic autism and the impact of TRPC6 disruption in human neurons	Mol Psychiatry	Use iPSCs from ASD patients	x					2D	Yes	Yes
2015	Human induced pluripotent stem cell derived neurons as a model for Williams-Beuren syndrome	Molecular Brain	Use iPSCs from a patient with ASD	x					2D	Yes	No
2015	FOXG1-Dependent Dysregulation of GABA/ Glutamate Neuron Differentiation in Autism Spectrum Disorders	Cell	Use iPSCs from ASD patients	x					Neural organoid	Yes	No
2015	CRISPR/Cas9-mediated heterozygous knockout of the autism gene CHD8 and characterization of its transcriptional networks in neurodevelopment	Mol Autism	Use a CRISPR/ Cas9-mediated CHD8 knockout-iPSCs - CHD8 is a gene strongly associated with ASD			x			2D	No	No
2016	Human Pluripotent Stem Cell-derived Cortical Neurons for High Throughput Medication Screening in Autism: A Proof of Concept Study in SHANK3 Haploinsufficiency Syndrome	EBioMedicine	Use iPSCs from ASD patients	x					2D	No	No
2016	Impaired Mitochondrial Dynamics and Mitophagy in Neuronal Models of Tuberous Sclerosis Complex	Cell Reports	Use iPSCs from Tuberous sclerosis complex (TSC) and ASD patients	x					2D	No	Yes
2016	Olfactory stem cells reveal MOCOS as a new player in autism spectrum disorders	Molecular Psychiatry	Use MOCOS-knockout-iPSCs - a gene associated with ASD			x			2D	No	Yes

continue...

...Continuation

Table 2S. Inclusion criteria for each selected article

Year	Title	Journal	Iclusion criteria	ASD	RTT	CTRL GM	ASD ENV	CTRL ENV	2D/3D	Electrophysiological studies	Use animals
2016	Collybistin binds and inhibits mTORC1 signaling: a potential novel mechanism contributing to intellectual disability and autism	Eur J Hum Genet	Use iPSCs from ASD patients	x					2D	No	Yes
2016	Altered neuronal network and rescue in a human MECP2 duplication model	Molecular Psychiatry	Use iPSCs from patients with MECP2 duplications and ASD	x					2D	Yes	Yes
2016	KCC2 rescues functional deficits in human neurons derived from patients with Rett syndrome	Proc Natl Acad Sci U S A	Use iPSCs from Ret Syndrome (RTT) patients - a disorder strongly associated with ASD		x				2D	Yes	Yes
2016	A rare inherited 15q 11.2-q13.1 interstitial duplication with maternal somatic mosaicism renal carcinoma, and autism	Frontiers in Genetics	Use iPSCs from patients with ASD	x					2D	No	No
2016	Establishment of EHMT1 mutant induced pluripotent stem cell (iPSC) line from a 11-year-old Kleefstra syndrome (KS) patient with autism and normal intellectual performance	Stem Cell Res	Use iPSCs from a Kleefstra syndrome (KS)/ ASD patient	x					2D	No	No
2017	Personalized genome sequencing coupled with iPSC technology identifies GTDC1 as a gene involved in neurodevelopmental disorders	Hum Mol Genet	Use iPSCs from an individual with chromossomal translocation that affects gene GTDC1 - The authors found that GTDC1 is a gene associated with ASD	x					2D	No	Yes
2017	Molecular analyses of neurogenic defects in a human pluripotent stem cell model of fragile X syndrome	Brain	Use iPSCs from Fragile X Syndrome/ASD patients	x					2D	Yes	No
2017	Psychosis Risk Candidate ZNF804A Localizes to Synapses and Regulates Neurite Formation and Dendritic Spine Structure	Biol Psychiatry	Use a ZNF804A knockdown-iPSCs - ZNF804A is a gene associated with ASD			x			2D	No	Yes
2017	Cellular Phenotypes in Human iPSC-Derived Neurons from a Genetic Model of Autism Spectrum Disorder	Cell Rep	Use iPSCs from ASD patients	x					2D	Yes	No
2017	Idiopathic Autism: Cellular and Molecular Phenotypes in Pluripotent Stem Cell-Derived Neurons	Mol Neurobiol	Use iPSCs from ASD patients	x					2D	Yes	Yes

continue...

...Continuation

Table 2S. Inclusion criteria for each selected article

Year	Title	Journal	Iclusion criteria	ASD	RTT	CTRL GM	ASD ENV	CTRL ENV	2D/3D	Electrophysiological studies	Use animals
2017	Altered proliferation and networks in neural cells derived from idiopathic autistic individuals	Mol Psychiatry	Use iPSCs from ASD patients	x					2D	Yes	Yes
2017	Altered neurite morphology and cholinergic function of induced pluripotent stem cell-derived neurons from a patient with Kleefstra syndrome and autism	Translational Psychiatry	Use iPSCs from a Kleefstra syndrome (KS)/ ASD patient	x					2D	Yes	No
2017	Zinc deficiency and low enterocyte zinc transporter expression in human patients with autism related mutations in SHANK3	Sci Rep	Use iPSCs from Phelan–McDermid syndrome (PMDS) - a syndrome strongly associated with ASD	x					2D	No	Yes
2017	Establishment of an induced pluripotent stem cell (iPSC) line from a 9-year old male with autism spectrum disorder (ASD)	Stem Cell Res	Use iPSCs from ASD patients	x					2D	No	No
2017	CRISPR/Cas9-mediated heterozygous knockout of the autism gene CHD8 and characterization of its transcriptional networks in cerebral organoids derived from iPS cells	Mol Autism	Use a CRISPR/Cas9-mediated CHD8+/− iPSCs - CHD8 is a gene associated with ASD			x			Neural organoid	No	No
2017	Mutations in RAB39B in individuals with intellectual disability, autism spectrum disorder, and macrocephaly	Mol Autism	Use CRISPR/Cas9-mediated RAB39B iPSCs - The authors found that RAB39B is a gene associated with ASD			x			2D	No	No
2017	Luteolin attenuates interleukin-6-mediated astrogliosis in human iPSC-derived neural aggregates: A candidate preventive substance for maternal immune activation-induced abnormalities	Neurosci Lett	Use IL-6 treated iPSCs to mimic a MIA model of ASD					x	Neural organoid	No	No
2018	Complete Disruption of Autism-Susceptibility Genes by Gene Editing Predominantly Reduces Functional Connectivity of Isogenic Human Neurons	Stem Cell Reports	Use CRISPR/Cas9 to KO 10 ASD-related genes in iPSCs-derived neurons			x			2D	Yes	Yes
2018	Convergent Pathways in Idiopathic Autism Revealed by Time Course Transcriptomic Analysis of Patient-Derived Neurons	Sci Rep	Use iPSCs from ASD patients	x					2D	Yes	No

continue...

...Continuation

Table 2S. Inclusion criteria for each selected article

Year	Title	Journal	Iclusion criteria	ASD	RTT	CTRL GM	ASD ENV	CTRL ENV	2D/3D	Electrophysiological studies	Use animals
2018	Actin cytoskeleton dynamics in stem cells from autistic individuals	Sci Rep	Use iPSCs from ASD patients	x					2D	No	No
2018	THC exposure of human iPSC neurons impacts genes associated with neuropsychiatric disorders	Transl Psychiatry	Use iPSCs from neurotypical and schizophrenic patients exposed to THC and found some altered genes related to ASD					x	2D	No	No
2018	Stem cell-derived neurons from autistic individuals with SHANK3 mutation show morphogenetic abnormalities during early development	Mol Psychiatry	Use iPSCs from ASD patients	x					2D	No	No
2018	A rare human CEP290 variant disrupts the molecular integrity of the primary cilium and impairs Sonic Hedgehog machinery	Sci Rep	Use iPSCs from ASD patients	x					2D	No	Yes
2018	MeCP2-regulated miRNAs control early human neurogenesis through differential effects on ERK and AKT signaling	Mol Psychiatry	Use iPSCs from Ret Syndrome (RTT) patients - a disorder strongly associated with ASD		x				Neural organoid	No	Yes
2018	Modeling the Interplay Between Neurons and Astrocytes in Autism Using Human Induced Pluripotent Stem Cells	Biol Psychiatry	Use iPSCs from ASD patients	x					2D	Yes	No
2018	Rare RELN variants affect Reelin-DAB1 signal transduction in autism spectrum disorder	Hum Mutat	Use iPSCs from ASD patients	x					2D	No	No
2018	Purkinje cells derived from TSC patients display hypoexcitability and synaptic deficits associated with reduced FMRP levels and reversed by rapamycin	Mol Psychiatry	Use iPSCs from Tuberous sclerosis complex (TSC)/ ASD patients	x					2D	Yes	Yes
2018	Single-molecule fluorescence in-situ hybridization reveals that human SHANK3 mRNA expression varies during development and in autism-associated SHANK3 heterozygosity	Stem Cell Res Ther	Use iPSCs from ASD patients	x					2D	No	Yes
2019	Dynamical Electrical Complexity Is Reduced during Neuronal Differentiation in Autism Spectrum Disorder	Stem Cell Reports	Use iPSCs from ASD patients	x					2D	Yes	No

continue...

...Continuation

Table 2S. Inclusion criteria for each selected article

Year	Title	Journal	Iclusion criteria	ASD	RTT	CTRL GM	ASD ENV	CTRL ENV	2D/3D	Electrophysiological studies	Use animals
2019	Increased Ca2+ signaling in NRXN1α +/- neurons derived from ASD induced pluripotent stem cells	Molecular Autism	Use iPSCs from ASD patients	x					2D	No	No
2019	Generation of seven iPSC lines from peripheral blood mononuclear cells suitable to investigate Autism Spectrum Disorder	Stem Cell Res	Use iPSCs from ASD patients	x					2D	No	No
2019	Haploinsufficiency in the ANKS1B gene encoding AIDA-1 leads to a neurodevelopmental syndrome	Nat Commun	Use iPSCs from ASD patients	x					2D	Yes	Yes
2019	mTOR and autophagy pathways are dysregulated in murine and human models of Schaaf-Yang syndrome	Sci Rep	Use iPSCs from patients with Schaaf-Yang syndrome (SHFYNG), a syndrome associated with ASD	x					2D	No	Yes
2019	CNTN5(-)(/+)or EHMT2(-)(/+)human iPSC-derived neurons from individuals with autism develop hyperactive neuronal networks	Elife	Use iPSCs from ASD patients	x					2D	Yes	Yes
2019	Derivation of familial iPSC lines from three ASD patients carrying NRXN1α(+/-) and two controls (NUIGi022-A, NUIGi022-B; NUIGi023-A, NUIGi023-B; NUIGi025-A, NUIGi025-B; NUIGi024-A, NUIGi024-B; NUIGi026-A, NUIGi026-B)	Stem Cell Res	Use iPSCs from ASD patients	x					2D	No	No
2019	Altered spinogenesis in iPSC-derived cortical neurons from patients with autism carrying de novo SHANK3 mutations	Sci Rep	Use iPSCs from ASD patients	x					2D	No	No
2019	Uncovering the Functional Link Between SHANK3 Deletions and Deficiency in Neurodevelopment Using iPSC-Derived Human Neurons	Front Neuroanat	Use SHANK3-nockdown-iPSCs - a condition strongly associated with ASD			x			2D	Yes	Yes
2019	Proteomic analyses reveal misregulation of LIN28 expression and delayed timing of glial differentiation in human iPS cells with MECP2 loss-of-function	PLoS One	Use iPSCs from Ret Syndrome (RTT) patients - a disorder strongly associated with ASD		x				2D	No	No

continue...

...Continuation

Table 2S. Inclusion criteria for each selected article

Year	Title	Journal	Iclusion criteria	ASD	RTT	CTRL GM	ASD ENV	CTRL ENV	2D/3D	Electrophysiological studies	Use animals
2019	Single cell analysis of autism patient with bi-allelic NRXN1-alpha deletion reveals skewed fate choice in neural progenitors and impaired neuronal functionality	Exp Cell Res	Use iPSCs from ASD patients	x					2D	Yes	No
2019	Cellular and molecular characterization of multiplex autism in human induced pluripotent stem cell-derived neurons	Mol Autism	Use iPSCs from ASD patients	x					2D	No	No
2019	Generation of iPSC line (GIBHi001-A) from a patient with autism spectrum disorder	Stem Cell Res	Use iPSCs from ASD patients	x					2D	No	Yes
2019	Downregulation of an Evolutionary Young miR-1290 in an iPSC-Derived Neural Stem Cell Model of Autism Spectrum Disorder	Stem Cells Int	Use iPSCs from ASD patients	x					2D	No	No
2019	Generation of an induced pluripotent stem cell line from a patient with autism spectrum disorder and SCN2A haploinsufficiency	Stem Cell Res	Use iPSCs from ASD patients	x					2D	No	No
2019	Pathological priming causes developmental gene network heterochronicity in autistic subject-derived neurons	Nat Neurosci	Use iPSCs from ASD patients	x					2D	Yes	No
2019	Control of Long-Term Synaptic Potentiation and Learning by Alternative Splicing of the NMDA Receptor Subunit GluN1	Cell Rep	Use iPSCs from ASD patients	x					2D	Yes	Yes
2019	Dysregulation of neuron differentiation in an autistic savant with exceptional memory	Mol Brain	Use iPSCs from ASD patients	x					2D	Yes	Yes
2019	An induced pluripotent stem cells line (SDQLCHi014-A) derived from urine cells of a patient with ASD and hyperactivity carrying a 303 kb de novo deletion at chr3p26.1 implicating GRM7 gene	Stem Cell Res	Use iPSCs from ASD patients	x					2D	No	Yes
2019	Generation of an induced pluripotent stem cell line (SDQLCHi008-A) from a patient with ASD and DD carrying an 830 kb de novo deletion at chr7q11.22 including the exon 1 of AUTS2 gene	Stem Cell Res	Use iPSCs from ASD patients	x					2D	No	Yes

continue...

...Continuation

Table 2S. Inclusion criteria for each selected article

Year	Title	Journal	Iclusion criteria	ASD	RTT	CTRL GM	ASD ENV	CTRL ENV	2D/3D	Electrophysiological studies	Use animals
2019	A pore-localizing CACNA1C-E1115K missense mutation, identified in a patient with idiopathic QT prolongation, bradycardia, and autism spectrum disorder, converts the L-type calcium channel into a hybrid nonselective monovalent cation channel	Heart Rhythm	Use iPSCs from a patient with ASD	x					2D	Yes	No
2019	SHANK2 mutations associated with autism spectrum disorder cause hyperconnectivity of human neurons	Nat Neurosci	Use iPSCs from ASD patients	x		x			2D	Yes	Yes
2020	Pharmacological intervention to restore connectivity deficits of neuronal networks derived from ASD patient iPSC with a TSC2 mutation	Mol Autism	Use iPSCs from ASD patients	x					2D	Yes	No
2020	Presynaptic dysfunction in CASK-related neurodevelopmental disorders	Translational Psychiatry	Use iPSCs from patients with ASD and mutations on the gene CASK	x					2D	No	No
2020	Insulin-like growth factor 1 partially rescues early developmental defects caused by SHANK2 knockdown in human neurons	Neural Regen Res	Use control iPSCs genetically modified with shSHANK2 - a condition associated with ASD			x			2D	Yes	Yes
2020	Generation of two hiPSC lines from a patient with autism spectrum disorder harboring a 120 kb deletion in SHANK2 and two control lines from each parent	Stem Cell Res	Use iPSCs from ASD patients	x					2D	No	No
2020	Neurodevelopmental impairment induced by prenatal valproic acid exposure shown with the human cortical organoid-on-a-chip model	Microsyst Nanoeng	Use iPSCs to study an envirmental etiology of ASD with Valproic Acid					x	Neural organoid	No	No
2020	Derivation of two iPSC lines from a sporadic ASD patient (NUIGi033-A) and a paternal control (NUIGi034-A)	Stem Cell Res	Use iPSCs from ASD patients	x					2D	No	No
2020	Generation of human induced pluripotent stem cells (SDUKIi002-A) from a 22-year-old male diagnosed with autism spectrum disorder	Stem Cell Res	Use iPSCs from ASD patients	x					2D	No	No

continue...

...Continuation

Table 2S. Inclusion criteria for each selected article

Year	Title	Journal	Iclusion criteria	ASD	RTT	CTRL GM	ASD ENV	CTRL ENV	2D/3D	Electrophysiological studies	Use animals
2020	Generation of autism spectrum disorder patient-derived iPSC line SDUKIi004-A	Stem Cell Res	Use iPSCs from ASD patients - Pervasive Developmental Disorder-Not Otherwise Specified (PDD-NOS)	x					2D	No	No
2020	Derivation of induced pluripotent stem cells (SDUKIi003-A) from a 20-year-old male patient diagnosed with Asperger syndrome	Stem Cell Res	Use iPSCs from ASD patients	x					2D	No	No
2020	Neuronal defects in a human cellular model of 22q11.2 deletion syndrome	Nat Med	Use iPSCs from 22q11.2 deletion syndrome (22q11DS) and ASD patients	x					Neural organoid	Yes	No
2020	Derivation of induced pluripotent stem cells (iPSCs) by retroviral transduction of skin fibroblasts from four patients suffering 7q11.23 microduplication syndrome	Stem Cell Research	Use iPSCs from patients with ASD and 7q11.23 microduplication syndrome	x					2D	No	Yes
2020	IGF-1 treatment causes unique transcriptional response in neurons from individuals with idiopathic autism	Mol Autism	Use iPSCs from ASD patients	x					2D	Yes	No
2020	SYNGAP1 Controls the Maturation of Dendrites, Synaptic Function, and Network Activity in Developing Human Neurons	J Neurosci	Use control iPSCs genetically modified with CRISPR/Cas9 for the gene SYNGAP1 - a condition associated with ASD			x			2D	Yes	Yes
2020	Autism-associated SHANK3 mutations impair maturation of neuromuscular junctions and striated muscles	Sci Transl Med	Use iPSCs from Phelan-McDermid syndrome (PMS) patients - a condition strongly associated with ASD			x			2D	No	Yes
2020	Assessment of developmental neurotoxicity induced by chemical mixtures using an adverse outcome pathway concept	Environ Health	Use controls iPSCs to test several chemicals to study neurodevelopmental disorders caused by environmental factors (including ASD)					x	2D	No	No
2020	Shifts in Ribosome Engagement Impact Key Gene Sets in Neurodevelopment and Ubiquitination in Rett Syndrome	Cell Rep	Use iPSCs from Ret Syndrome (RTT) patients - a disorder strongly associated with ASD		x				2D	No	Yes
2020	Synaptic Dysfunction in Human Neurons With Autism-Associated Deletions in PTCHD1-AS	Biol Psychiatry	Use iPSCs from ASD patients	x					2D	Yes	Yes

continue...

...Continuation
Table 2S. Inclusion criteria for each selected article

Year	Title	Journal	Iclusion criteria	ASD	RTT	CTRL GM	ASD ENV	CTRL ENV	2D/3D	Electrophysiological studies	Use animals
2020	16p11.2 microdeletion imparts transcriptional alterations in human iPSC-derived models of early neural development	Elife	Use iPSCs of patients with ASD and 16p11.2 copy number variations (CNVs)	x					2D	No	No
2020	Transcriptional consequences of MBD5 disruption in mouse brain and CRISPR-derived neurons	Mol Autism	Use a CRISPR edited MBD5-iPSCs - a condition associated with ASD			x			2D	No	Yes
2020	Integrative Analysis Identifies Key Molecular Signatures Underlying Neurodevelopmental Deficits in Fragile X Syndrome	Biol Psychiatry	Use FMR1-knockout-iPSCs by CRISPR/Cas9 edition - a condition strongly associated with ASD			x			2D	Yes	No
2020	Quantitative proteomic analysis of Rett iPSC-derived neuronal progenitors	Mol Autism	Use iPSCs from Ret Syndrome (RTT) patients - a disorder strongly associated with ASD		x				2D	No	No
2020	A chimeric mouse model to study human iPSC-derived neurons: the case of a truncating SHANK3 mutation	Sci Rep	Use iPSCs from ASD patients	x					2D	No	Yes
2020	Increased Neural Progenitor Proliferation in a hiPSC Model of Autism Induces Replication Stress-Associated Genome Instability	Cell Stem Cell	Use iPSCs from ASD patients	x					2D	No	No
2020	Interferon-γ signaling in human iPSC-derived neurons recapitulates neurodevelopmental disorder phenotypes	Sci Adv	Use iPSCs from ASD patients	x					2D	No	No
2020	Generation of Induced Pluripotent Stem Cells from a Female Patient with a Xq27.3-q28 Deletion to Establish Disease Models and Identify Therapies	Cellular Reprogramming	Use iPSCs from a patient with ASD and with a Xq27.3-q28 Deletion	x					2D	No	No
2020	Autism-associated PTEN missense mutation leads to enhanced nuclear localization and neurite outgrowth in an induced pluripotent stem cell line	FEBS J	Use control iPSCs genetically modified with CRISPR/Cas9 for the gene PTEN - a gene associated with ASD			x			2D	No	No
2020	Autism-associated variants of neuroligin 4X impair synaptogenic activity by various molecular mechanisms	Mol Autism	Use NL4X-knockout-iPSCs - a condition strongly associated with ASD (CRISPR-edited)			x			2D	No	Yes
2020	Cerebral organoid and mouse models reveal a RAB39b-PI3K-mTOR pathway-dependent dysregulation of cortical development leading to macrocephaly/autism phenotypes	Genes Dev	Use control iPSCs genetically modified with CRISPR/Cas9 for the gene RAB39b - a gene associated with ASD			x			Neural organoid	No	Yes

continue...

...Continuation

Table 2S. Inclusion criteria for each selected article

Year	Title	Journal	Iclusion criteria	ASD	RTT	CTRL GM	ASD ENV	CTRL ENV	2D/3D	Electrophysiological studies	Use animals
2021	Atypical Neurogenesis in Induced Pluripotent Stem Cells From Autistic Individuals	Biol Psychiatry	Use iPSCs from ASD patients	x					2D	No	No
2021	NRXN1α(+/-) is associated with increased excitability in ASD iPSC-derived neurons	BMC Neurosci	Use iPSCs from ASD patients	x					2D	Yes	No
2021	Integrated RNA Sequencing Reveals Epigenetic Impacts of Diesel Particulate Matter Exposure in Human Cerebral Organoids	Developmental Neuroscience	Use iPSCs from ASD patients to study an envirmental etiology of ASD with diesel particulate matter (DPM)	x			x	x	Neural organoid	No	No
2021	RHOA signaling defects result in impaired axon guidance in iPSC-derived neurons from patients with tuberous sclerosis complex	Nat Commun	Use iPSCs from Tuberous sclerosis complex (TSC) patients - a disorder associated with ASD	x		x			2D	No	No
2021	Defective AMPA-mediated synaptic transmission and morphology in human neurons with hemizygous SHANK3 deletion engrafted in mouse prefrontal cortex	Molecular Psychiatry	Use control iPSCs genetically modified with CRISPR/Cas9 for the gene SHANK3 - a gene strongly associated with ASD			x			2D	Yes	Yes
2021	Cortical overgrowth in a preclinical forebrain organoid model of CNTNAP2-associated autism spectrum disorder	Nat Commun	Use iPSCs from ASD patients	x					Neural organoid	No	No
2021	Generation of an iPSC line (CRICKi001-A) from an individual with a germline SMARCA4 missense mutation and autism spectrum disorder	Stem Cell Res	Use iPSCs from ASD patients	x					2D	No	No
2021	Derivation of iPSC lines from two patients with autism spectrum disorder carrying NRXN1α deletion (NUIGi041-A, NUIG041-B; NUIGi045-A) and one sibling control (NUIGi042-A, NUIGi042-B)	Stem Cell Res	Use iPSCs from ASD patients	x					2D	No	No
2021	Derivation of four iPSC lines from a male ASD patient carrying a deletion in the middle coding region of NRXN1α gene (NUIGi039-A and NUIGi039-B) and a male sibling control (NUIGi040-A and NUIGi040-B)	Stem Cell Res	Use iPSCs from ASD patients	x					2D	No	No

continue...

...Continuation

Table 2S. Inclusion criteria for each selected article

Year	Title	Journal	Iclusion criteria	ASD	RTT	CTRL GM	ASD ENV	CTRL ENV	2D/3D	Electrophysiological studies	Use animals
2021	Human induced pluripotent stem cell line (QBRIi013-A) derivation from a 6-year-old female diagnosed with Autism spectrum disorder (ASD) and intellectual disability (ID)	Stem Cell Res	Use iPSCs from ASD patients	x					2D	No	No
2021	Postnatal expression profiles of atypical cadherin FAT1 suggest its role in autism	Biol Open	Use iPSCs from ASD patients	x					2D	No	Yes
2021	Regulation of Neural Circuit Development by Cadherin-11 Provides Implications for Autism	eNeuro	Use iPSCs from ASD patients	x					2D	Yes	Yes
2021	Transcriptome of iPSC-derived neuronal cells reveals a module of co-expressed genes consistently associated with autism spectrum disorder	Mol Psychiatry	Use iPSCs from ASD patients	x					2D	No	No
2021	Derivation of iPSC lines from two idiopathic ASD patients (OFi001-A, OFi002-A)	Stem Cell Res	Use iPSCs from ASD patients	x					2D	No	Yes
2021	Establishment of an induced pluripotent stem (iPS) cell line (SDUKIi006-A) from a 21-year old male patient diagnosed with atypical autism disorder	Stem Cell Res	Use iPSCs from ASD patients	x					2D	No	No
2021	A human forebrain organoid model of fragile X syndrome exhibits altered neurogenesis and highlights new treatment strategies	Nature Neuroscience	Use iPSCs from patients with Fragile X syndrome (FXS) and found differential expression of genes related to ASD			x			Neural organoid	Yes	Yes
2021	Extracellular LGALS3BP regulates neural progenitor position and relates to human cortical complexity	Nature Communications	Use iPSCs from controls genome edited for the gene LGALS3BP to mimic a mutation on a patient with ASD	x		x			Neural organoid	No	Yes
2021	Overexpression of CD47 is associated with brain overgrowth and 16p11.2 deletion syndrome	Proc Natl Acad Sci U S A	They used hiPSCs derived from controls and subjects with ASD and 16p11.2 deletion syndrome	x					2D	No	Yes
2021	Dysfunction of NMDA receptors in neuronal models of an autism spectrum disorder patient with a DSCAM mutation and in Dscam-knockout mice	Mol Psychiatry	Use iPSCs from ASD patients	x					2D	Yes	Yes

continue...

...Continuation

Table 2S. Inclusion criteria for each selected article

Year	Title	Journal	Iclusion criteria	ASD	RTT	CTRL GM	ASD ENV	CTRL ENV	2D/3D	Electrophysiological studies	Use animals
2021	SHANK2 Mutations Result in Dysregulation of the ERK1/2 Pathway in Human Induced Pluripotent Stem Cells-Derived Neurons and Shank2(-/-) Mice	Front Mol Neurosci	Use iPSCs from ASD patients	x					2D	No	Yes
2021	Complement C4 Is Reduced in iPSC-Derived Astrocytes of Autism Spectrum Disorder Subjects	Int J Mol Sci	Use iPSCs from ASD patients	x					2D	No	No
2021	Altered neuronal physiology, development, and function associated with a common chromosome 15 duplication involving CHRNA7	BMC Biol	Use iPSCs from ASD patients	x					2D	Yes	Yes
2021	Gene-Environment Interactions in Developmental Neurotoxicity: a Case Study of Synergy between Chlorpyrifos and CHD8 Knockout in Human BrainSpheres	Environ Health Perspect	Use control iPSCs genetically modified with CRISPR/Cas9 for the gene CHD8- a gene strong associated with ASD			x			Neural organoid	No	No
2021	Transcriptome analysis of MBD5-associated neurodevelopmental disorder (MAND) neural progenitor cells reveals dysregulation of autism-associated genes	Sci Rep	Use iPSCs from ASD patients	x					2D	No	No
2021	5'-utr snp of fgf13 causes translational defect and intellectual disability	eLife	Use iPSCs from patients suspected of mild ASD and intellectual disability	x		x			2D	No	Yes
2021	CB1 antagonism increases excitatory synaptogenesis in a cortical spheroid model of fetal brain development	Scientific Reports	Use iPSCs from ASD patients	x					Neural organoid	Yes	No
2021	Combining *in vitro* assays and mathematical modelling to study developmental neurotoxicity induced by chemical mixtures	Reproductive Toxicology	Use six chemicals treated iPSCs to mimic environmental factors related to ASD					x	2D	Yes	No
2021	Impaired expression of the COSMOC/MOCOS gene unit in ASD patient stem cells	Mol Psychiatry	Use control iPSCs genetically modified with CRISPR for COSMOC genes, and found relation to ASD			x			2D	No	No
2021	16p11.2 deletion is associated with hyperactivation of human iPSC-derived dopaminergic neuron networks and is rescued by RHOA inhibition *in vitro*	Nature Communications	Use control iPSCs genetically modified with CRISPR for 16p11.2del and 16p11.2dup, and found relation to ASD genes			x			2D	Yes	No

continue...

...Continuation

Table 2S. Inclusion criteria for each selected article

Year	Title	Journal	Iclusion criteria	ASD	RTT	CTRL GM	ASD ENV	CTRL ENV	2D/3D	Electrophysiological studies	Use animals
2021	Cortical organoids model early brain development disrupted by 16p11.2 copy number variants in autism	Mol Psychiatry	Use iPSCs from ASD patients	x					Neural organoid	No	No
2021	Parallel in vivo analysis of large-effect autism genes implicates cortical neurogenesis and estrogen in risk and resilience	NEURON	Use control iPSCs genetically modified with CRISPR for several ASD genes			x			Neural organoid	No	Yes
2021	Network Effects of the 15q13.3 Microdeletion on the Transcriptome and Epigenome in Human-Induced Neurons	Biol Psychiatry	Use iPSCs from patients with ASD and 15q13.3 microdeletions. Also, used iPSCs genetically modified for several ASD-related genes	x		x			2D	No	No
2022	Generation of induced pluripotent stem cell lines from nonaffected parents and monozygotic triplets affected with autism spectrum disorder and epilepsy	Stem Cell Res	Use iPSCs from ASD patients	x					2D	No	No
2022	Astrocytes derived from ASD individuals alter behavior and destabilize neuronal activity through aberrant Ca2+ signaling	Molecular Psychiatry	Use iPSCs from a patient with ASD	x					Neural organoid	Yes	Yes
2022	NRSF/REST lies at the intersection between epigenetic regulation, miRNA-mediated gene control and neurodevelopmental pathways associated with Intellectual disability (ID) and Schizophrenia	Translational Psychiatry	Use iPSCs from a patient with ASD	x					2D	No	Yes
2022	16p13.11 deletion variants associated with neuropsychiatric disorders cause morphological and synaptic changes in induced pluripotent stem cell-derived neurons	Front Psychiatry	Use iPSCs from ASD patients	x					2D	No	No
2022	From cohorts to molecules: Adverse impacts of endocrine disrupting mixtures	Science	Use control iPSCs to model an environmental model of ASD with Endocrine disrupting chemicals (EDCs)					x	Neural organoid	No	Yes
2022	Generation of an induced pluripotent stem cell line SJTUXHi001-A from an autism spectrum disorder patient carrying a heterozygous mutation in HDAC8 (p.P359S)	Stem Cell Res	Use iPSCs from ASD patients	x					2D	No	Yes

continue...

...Continuation

Table 2S. Inclusion criteria for each selected article

Year	Title	Journal	Iclusion criteria	ASD	RTT	CTRL GM	ASD ENV	CTRL ENV	2D/3D	Electrophysiological studies	Use animals
2022	Autism NPCs from both idiopathic and CNV 16p11.2 deletion patients exhibit dysregulation of proliferation and mitogenic responses	Stem Cell Reports	Use iPSCs from ASD patients	x					2D	No	No
2022	Multi-Omic Investigations of a 17-19 Translocation Links MINK1 Disruption to Autism, Epilepsy and Osteoporosis	Int J Mol Sci	Use iPSCs from ASD patients	x					2D	No	No
2022	Superoxide dismutase isozymes in cerebral organoids from autism spectrum disorder patients	J Neural Transm (Vienna)	Use iPSCs from ASD patients	x					Neural organoid	No	No
2022	Interleukin-17a Induces Neuronal Differentiation of Induced-Pluripotent Stem Cell-Derived Neural Progenitors From Autistic and Control Subjects	Front Neurosci	Use iPSCs from ASD patients	x			x	x	2D	No	No
2022	Generation of a MIR5004 knockout cell line from human induced pluripotent stem cells by CRISPR/Cas9 gene editing	Stem Cell Res	Use control iPSCs genetically modified with CRISPR for ASD risk gene MIR5004/SYNGAP1			x			2D	No	No
2022	Generation of a human induced pluripotent stem cell line (SJTUXHi002-A) from an individual with autism spectrum disorder carrying a heterozygous mutation in GRIA2	Stem Cell Res	Use iPSCs from ASD patients	x					2D	No	Yes
2022	CHD8 suppression impacts on histone H3 lysine 36 trimethylation and alters RNA alternative splicing	Nucleic Acids Res	Use control iPSCs genetically modified for ASD risk gene CHD8			x			2D	No	No
2022	Loss of function of OTUD7A in the schizophrenia- associated 15q13.3 deletion impairs synapse development and function in human neurons	Am J Hum Genet	Use control iPSCs genetically modified with CRISPR for the gene OTUD7A and found relation to ASD risk genes			x			2D	Yes	Yes
2022	Orgo-Seq integrates single-cell and bulk transcriptomic data to identify cell type specific-driver genes associated with autism spectrum disorder	Nat Commun	Use iPSCs from ASD patients	x					Neural organoid	No	No
2022	SHANK3 deficiency leads to myelin defects in the central and peripheral nervous system	Cell Mol Life Sci	Use iPSCs from patients with Phelan–McDermid syndrome (PMDS), as a syndromic form of autism spectrum disorders (ASDs)			x			Neural organoid	No	Yes

continue...

...Continuation

Table 2S. Inclusion criteria for each selected article

Year	Title	Journal	Iclusion criteria	ASD	RTT	CTRL GM	ASD ENV	CTRL ENV	2D/3D	Electrophysiological studies	Use animals
2022	Human forebrain organoids reveal connections between valproic acid exposure and autism risk	Transl Psychiatry	Use control iPSCs to model an environmental model of ASD with Valproic acid (VPA) exposure					x	Neural organoid	Yes	No
2022	Wide spectrum of neuronal and network phenotypes in human stem cell-derived excitatory neurons with Rett syndrome-associated MECP2 mutations	Transl Psychiatry	Use iPSCs from Rett Syndrome patients		x	x			2D	Yes	Yes
2022	Neuroprogenitor Cells From Patients With TBCK Encephalopathy Suggest Deregulation of Early Secretory Vesicle Transport	Frontiers in Cellular Neuroscience	Use iPSCs from a patient with ASD	x					2D	No	No
2022	Neuron-specific protein network mapping of autism risk genes identifies shared biological mechanisms and disease-relevant pathologies	Cell Reports	Use control iPSCs genetically edited for the ASD risk gene TAOK2			x			2D	Yes	Yes
2022	The 22q11.2 region regulates presynaptic gene-products linked to schizophrenia	Nat Commun	Use iPSCs from patients with 22q11.2del and ASD	x					2D	Yes	Yes
2022	Autism genes converge on asynchronous development of shared neuron classes	Nature	Use control iPSCs genetically modified with CRISPR for three ASD genes (SUV420H1, ARID1B and CHD8)			x			Neural organoid	Yes	No
2022	Interferon-γ exposure of human iPSC-derived neurons alters major histocompatibility complex I and synapsin protein expression	Front Psychiatry	Use control iPSCs to model an environmental model of ASD with to interferon-Y exposure					x	2D	No	No
2022	DNA Methylation Profiles of GAD1 in Human Cerebral Organoids of Autism Indicate Disrupted Epigenetic Regulation during Early Development	Int J Mol Sci	Use iPSCs from ASD patients	x					Neural organoid	No	No
2022	Screening for modulators of autism spectrum disorder using induced human neurons	SLAS Discovery	Use control iPSCs from an ASD individual and genetically modified iPSCs with CRISPR for four ASD risk genes (ADNP, FOXP1, DDX3X, SHANK3)	x		x			2D	No	No
2022	Cell line specific alterations in genes associated with dopamine metabolism and signaling in midbrain dopaminergic neurons derived from 22q11.2 deletion carriers with elevated dopamine synthesis capacity	Schizophr Res	Use iPSCs from ASD patients	x					2D	No	No

continue...

...Continuation

Table 2S. Inclusion criteria for each selected article

Year	Title	Journal	Iclusion criteria	ASD	RTT	CTRL GM	ASD ENV	CTRL ENV	2D/3D	Electrophysiological studies	Use animals
2022	Aberrant induction of p19Arf-mediated cellular senescence contributes to neurodevelopmental defects	PLoS Biol	Use control iPSCs to model an environmental model of ASD with Valproic acid (VPA) exposure					x	Neural organoid	No	Yes
2022	Tissue- and cell-type-specific molecular and functional signatures of 16p11.2 reciprocal genomic disorder across mouse brain and human neuronal models	Am J Hum Genet	Use control iPSCs genetically modified with CRISPR for 16p11.2del and 16p11.2dup, and found relation to ASD genes			x			Neural organoid	Yes	Yes
2022	Rare CACNA1H and RELN variants interact through mTORC1 pathway in oligogenic autism spectrum disorder	Transl Psychiatry	Use iPSCs from ASD patients	x					2D	No	No
2022	Krüppel-like Transcription Factor 7 Is a Causal Gene in Autism Development	Int J Mol Sci	Use control iPSCs genetically modified for the ASD risk genes (klf7)			x			Neural organoid	No	Yes
2022	Generation of the human induced pluripotent stem cell line (IBKMOLi002-A) from PBMCs of a patient carrying the heterozygous L271H mutation of the voltage-gated calcium channel subunit Ca(v)1.3-encoding CACNA1D gene	Stem Cell Res	Use iPSCs from ASD patients	x					2D	No	No
2022	Neuronal hyperexcitability and ion channel dysfunction in CDKL5-deficiency patient iPSC-derived cortical organoids	Neurobiol Dis	Use iPSCs from Rett Syndrome and CDKL5-deficiency disorder (CDD) patients *Rodent in silico analyses		x				Neural organoid	Yes	No
2022	miR-92a-2-5p Regulates the Proliferation and Differentiation of ASD-Derived Neural Progenitor Cells	Current Issues in Molecular Biology	Use iPSCs from a patient with ASD	x					2D	No	No
2023	Screening autism-associated environmental factors in differentiating human neural progenitors with fractional factorial design-based transcriptomics	Sci Rep	Use iPSCs from ASD patients	x			x	x	2D	No	No
2023	Non-synaptic function of the autism spectrum disorder-associated gene SYNGAP1 in cortical neurogenesis	Nat Neurosci	Use control iPSCs genetically edited for the ASD risk gene SYNGAP1			x			Neural organoid	No	Yes
2023	Disruption of DDX53 coding sequence has limited impact on iPSC-derived human NGN2 neurons	BMC Med Genomics	Use control iPSCs genetically edited for the ASD risk gene DDX53			x			2D	Yes	No

continue...

...Continuation

Table 2S. Inclusion criteria for each selected article

Year	Title	Journal	Iclusion criteria	ASD	RTT	CTRL GM	ASD ENV	CTRL ENV	2D/3D	Electrophysiological studies	Use animals
2023	Generation and characterization of PBMCs-derived human induced pluripotent stem cell (iPSC) line SDQLCHi051-A from an autism spectrum disorder patient with compound CHD8 gene mutations	Stem Cell Res	Use iPSCs from ASD patients	x					2D	No	No
2023	Modeling maternal cholesterol exposure reveals a reduction of neural progenitor proliferation using human cerebral organoids	Life Medicine	Use control iPSCs to model an environmental model of ASD with cholesterol exposure					x	Neural organoid	No	No
2023	16p11.2 deletion accelerates subpallial maturation and increases variability in human iPSC-derived ventral telencephalic organoids	Development	Use control iPSCs genetically edited for the ASD CNV 16p11.2			x			Neural organoid	No	No
2023	Autism-specific PTEN p.Ile135Leu variant and an autism genetic background combine to dysregulate cortical neurogenesis	Am J Hum Genet	Use iPSCs from ASD patients	x					Neural organoid	No	No
2023	Novel Calmodulin Variant p.E46K Associated With Severe Catecholaminergic Polymorphic Ventricular Tachycardia Produces Robust Arrhythmogenicity in Human Induced Pluripotent Stem Cell-Derived Cardiomyocytes	Circulation: Arrhythmia and Electrophysiology	Use iPSCs from ASD patients	x					2D	Yes	No
2023	Gain and loss of function variants in EZH1 disrupt neurogenesis and cause dominant and recessive neurodevelopmental disorders	Nature Communications	Use control iPSCs genetically edited for the ASD risk gene EZH1			x			Neural organoid	No	Yes
2023	Early maturation and hyperexcitability is a shared phenotype of cortical neurons derived from different ASD-associated mutations	Transl Psychiatry	Use iPSCs from ASD patients	x					2D	Yes	No
2023	Modeling idiopathic autism in forebrain organoids reveals an imbalance of excitatory cortical neuron subtypes during early neurogenesis	Nat Neurosci	Use iPSCs from ASD patients	x					Neural organoid	No	No
2023	PLPPR4 haploinsufficiency causes neurodevelopmental disorders by disrupting synaptic plasticity via mTOR signalling	Journal of Cellular and Molecular Medicine	Use iPSCs from ASD patients	x					2D	No	No

continue...

...Continuation

Table 2S. Inclusion criteria for each selected article

Year	Title	Journal	Iclusion criteria	ASD	RTT	CTRL GM	ASD ENV	CTRL ENV	2D/3D	Electrophysiological studies	Use animals
2023	Deficiency of the Heterogeneous Nuclear Ribonucleoprotein U locus leads to delayed hindbrain neurogenesis	Biology Open	Use iPSCs from ASD patients	x					2D	Yes	No
2023	Transcriptional Dysregulation and Impaired Neuronal Activity in FMR1 Knock-Out and Fragile X Patients' iPSC-Derived Models	International Journal of Molecular Sciences	Use control iPSCs genetically edited for the ASD risk gene FMR1			x			2D	Yes	No
2023	FOXP1 orchestrates neurogenesis in human cortical basal radial glial cells	PLoS Biol	Use control iPSCs genetically edited for the ASD risk gene FOXP1			x			Neural organoid	No	No
2023	CAPRIN1 haploinsufficiency causes a neurodevelopmental disorder with language impairment, ADHD and ASD	Brain	Use control iPSCs genetically edited for the ASD risk gene CAPRIN1			x			2D	Yes	No
2023	Cell-type specific defects in PTEN-mutant cortical organoids converge on abnormal circuit activity	Hum Mol Genet	Use control iPSCs genetically edited for the ASD risk gene PTEN			x			Neural organoid	No	No
2023	Protein interaction studies in human induced neurons indicate convergent biology underlying autism spectrum disorders	Cell Genom	Use control iPSCs genetically edited for the ASD risk gene ANK2			x			2D	No	Yes
2023	Generation of induced pluripotent stem cell lines from three individuals with autism spectrum disorder	Stem Cell Res	Use iPSCs from ASD patients	x					2D	No	No
2023	Human brain organoid model of maternal immune activation identifies radial glia cells as selectively vulnerable	Mol Psychiatry	Use control iPSCs to model an environmental model of ASD with hyper-IL6 exposure					x	Neural organoid	No	No
2023	An *in vivo* neuroimmune organoid model to study human microglia phenotypes	Cell	Use iPSCs from ASD patients	x					Neural organoid	No	Yes
2023	Deletion of TRPC6, an Autism Risk Gene, Induces Hyperexcitability in Cortical Neurons Derived from Human Pluripotent Stem Cells	Mol Neurobiol	Use control iPSCs genetically edited for the ASD risk gene TRPC6			x			2D	Yes	No
2023	ANK2 loss-of-function variants are associated with epilepsy, and lead to impaired axon initial segment plasticity and hyperactive network activity in hiPSC-derived neuronal networks	Hum Mol Genet	Use control iPSCs genetically edited for the ASD risk gene ANK2			x			2D	Yes	Yes

continue...

...Continuation
Table 2S. Inclusion criteria for each selected article

Year	Title	Journal	Iclusion criteria	ASD	RTT	CTRL GM	ASD ENV	CTRL ENV	2D/3D	Electrophysiological studies	Use animals
2023	The distribution of neuroligin4, an autism-related postsynaptic molecule, in the human brain	Molecular Brain	Use control iPSCs genetically edited for the ASD risk gene NLGN4X			x			2D	No	No
2023	Impaired OTUD7A-dependent Ankyrin regulation mediates neuronal dysfunction in mouse and human models of the 15q13.3 microdeletion syndrome	Mol Psychiatry	Use iPSCs from ASD patients	x					2D	Yes	Yes
2023	Generation of an induced pluripotent stem cell line (IPCASi001-A) from an autism spectrum disorder individual without intellectual disability	Stem Cell Res	Use iPSCs from ASD patients	x					2D	No	No
2024	Characterization of a loss-of-function NSF attachment protein beta mutation in monozygotic triplets affected with epilepsy and autism using cortical neurons from proband-derived and CRISPR-corrected induced pluripotent stem cell lines	Front Neurosci	Use iPSCs from ASD patients	x					2D	Yes	No
2024	Molecular and network disruptions in neurodevelopment uncovered by single cell transcriptomics analysis of CHD8 heterozygous cerebral organoids	Heliyon	Use control iPSCs genetically edited for the ASD risk genes CHD8			x			Neural organoid	No	No
2024	Zika virus vertical transmission induces neuroinflammation and synapse impairment in brain cells derived from children born with Congenital Zika Syndrome	Sci Rep	Use iPSCs from ASD patients	x					2D	No	No
2024	Bi-allelic NRXN1α deletion in microglia derived from iPSC of an autistic patient increases interleukin-6 production and impairs supporting function on neuronal networking	Brain Behav Immun	Use iPSCs from ASD patients	x					2D	Yes	No
2024	Disruption of the autism-associated gene SCN2A alters synaptic development and neuronal signaling in patient iPSC-glutamatergic neurons	Front Cell Neurosci	Use iPSCs from ASD patients	x					2D	Yes	Yes

continue...

...Continuation

Table 2S. Inclusion criteria for each selected article

Year	Title	Journal	Iclusion criteria	ASD	RTT	CTRL GM	ASD ENV	CTRL ENV	2D/3D	Electrophysiological studies	Use animals
2024	Neuronal lineage tracing from progenitors in human cortical organoids reveals mechanisms of neuronal production, diversity, and disease	Cell Rep	Use iPSCs from ASD patients	x					Neural organoid	No	No
2024	Embryonic origin of two ASD subtypes of social symptom severity: the larger the brain cortical organoid size, the more severe the social symptoms	Mol Autism	Use iPSCs from ASD patients	x					Neural organoid	No	No
2024	Human cytomegalovirus (CMV) dysregulates neurodevelopmental pathways in cerebral organoids	Commun Biol	Use control iPSCs to model an environmental model of ASD with cytomegalovirus (CMV) infection					x	Neural organoid	No	No
2024	CRISPR-Cas9-generated PTCHD1 2489T>G stem cells recapitulate patient phenotype when undergoing neural induction	HGG Adv	Use control iPSCs genetically edited for the ASD risk genes PTCHD1			x			2D	No	No
2024	MARK2 variants cause autism spectrum disorder via the downregulation of WNT/β-catenin signaling pathway	Am J Hum Genet	Use iPSCs from ASD patients	x					2D	No	Yes
2024	Autism patient-derived SHANK2B(Y29X) mutation affects the development of ALDH1A1 negative dopamine neuron	Mol Psychiatry	Use iPSCs from ASD patients	x					2D	Yes	Yes
2024	Establishment of a non-integrated iPS cell line (SDQLCHi072-A) from a patient suffered from AUTS2 syndrome	Stem Cell Research	Use iPSCs from ASD patients	x					2D	No	No
2024	Generation of nine induced pluripotent stem cell lines from six young children with autism spectrum disorder and three matched control subjects from the Qatari population	Stem Cell Res	Use iPSCs from ASD patients	x					2D	No	No
2024	iPSC-Derived Astrocytes and Neurons Replicate Brain Gene Expression, Epigenetic, Cell Morphology and Connectivity Alterations Found in Autism	Cells	Use iPSCs from ASD patients	x					2D	No	No

continue...

...Continuation
Table 2S. Inclusion criteria for each selected article

Year	Title	Journal	Iclusion criteria	ASD	RTT	CTRL GM	ASD ENV	CTRL ENV	2D/3D	Electrophysiological studies	Use animals
2024	Zika virus infection impairs synaptogenesis, induces neuroinflammation, and could be an environmental risk factor for autism spectrum disorder outcome	Biochim Biophys Acta Mol Basis Dis	Use control iPSCs to model an environmental model of ASD with Zika virus exposure					x	2D	No	Yes
2024	Dysregulation of mTOR signaling mediates common neurite and migration defects in both idiopathic and 16p11.2 deletion autism neural precursor cells	Elife	Use iPSCs from ASD patients	x					2D	No	No
2024	Neural and metabolic dysregulation in PMM2-deficient human *in vitro* neural models	Cell Reports	Use iPSCs from ASD patients	x					Neural organoid	Yes	No
2024	Generation of an induced pluripotent stem cell line GWCMCi006-A from a patient with autosomal dominant neurodevelopmental disorder with or without hyperkinetic movements and seizures harboring GRIN1 c.389A > G mutation	Stem Cell Research	Use iPSCs from ASD patients	x					2D	No	No
2024	Dysregulation of miRNA expression and excitation in MEF2C autism patient hiPSC-neurons and cerebral organoids	Mol Psychiatry	Use iPSCs from ASD patients	x		x			Neural organoid	Yes	Yes
2024	Post-transcriptional dysregulation in autism, schizophrenia, and bipolar disorder	J Biomed Res	Use control iPSCs genetically edited for the ASD risk gene ELAVL3			x			Neural organoid	No	No
2024	Generation and transcriptomic characterization of MIR137 knockout miniature pig model for neurodevelopmental disorders	Cell and Bioscience	Use control iPSCs genetically edited for the ASD risk genes MIR137			x			2D	No	Yes

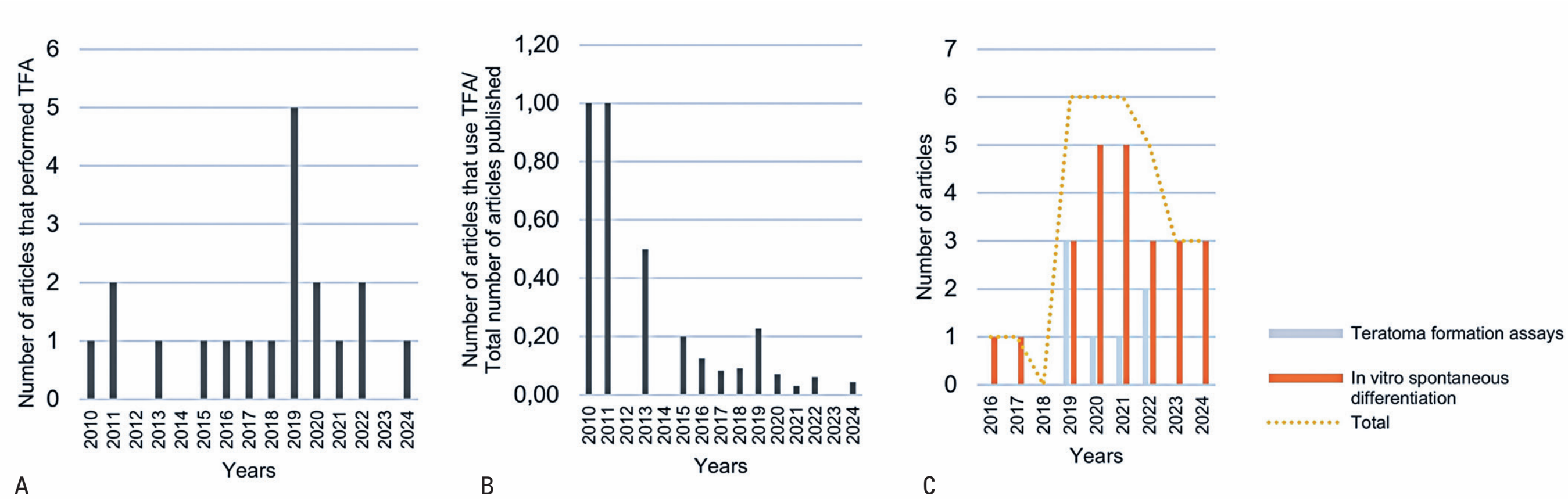

Figure 1S. Use of immunodeficient animals for performing TFA. A) The graph shows the number of studies that used immunodeficient mice to evaluate the pluripotency of hiPSCs by a TFA. B) The graph shows the proportion of studies that used immunodeficient mice to perform TFA relative to the total number of studies using hiPSCs in ASD research. C) The graph shows the number of studies using spontaneous differentiation through EB formation and animals for TFA

Table 3S. Use of immunodeficient mice in teratoma formation assays to validate hiPSC pluripotency across reviewed studies

#	Author	Year	Title	Journal	Teratoma formation assays	Mouse strains
399	Marchetto MC, Carromeu C, Acab A, Yu D, Yeo GW, Mu Y, Chen G, Gage FH, Muotri AR.	2010	A model for neural development and treatment of Rett syndrome using human induced pluripotent stem cells	Cell	Yes	CByJ.Cg-Foxn1nu/J
382	Ananiev G, Williams EC, Li H, Chang Q.	2011	Isogenic pairs of wild type and mutant induced pluripotent stem cell (iPSC) lines from Rett syndrome patients as *in vitro* disease model	PLoS One	Yes	NOD/SCID
463	Cheung AY, Horvath LM, Grafodatskaya D, Pasceri P, Weksberg R, Hotta A, Carrel L, Ellis J.	2011	Isolation of MECP2-null Rett Syndrome patient hiPS cells and isogenic controls through X-chromosome inactivation	Hum Mol Genet	Yes	Not clear
443	Zeng L, Zhang P, Shi L, Yamamoto V, Lu W, Wang K.	2013	Functional impacts of NRXN1 knockdown on neurodevelopment in stem cell models	PLoS One	Yes	Not clear
347	Griesi-Oliveira K, Acab A, Gupta AR, Sunaga DY, Chailangkarn T, Nicol X, Nunez Y, Walker MF, Murdoch JD, Sanders SJ, Fernandez TV, Ji W, Lifton RP, Vadasz E, Dietrich A, Pradhan D, Song H, Ming GL, Gu X, Haddad G, Marchetto MC, Spitzer N, Passos-Bueno MR, State MW, Muotri AR.	2015	Modeling non-syndromic autism and the impact of TRPC6 disruption in human neurons	Mol Psychiatry	Yes	Not clear
1125	Nageshappa S.; Carromeu C.; Trujillo C.A.; Mesci P.; Espuny-Camacho I.; Pasciuto E.; Vanderhaeghen P.; Verfaillie C.M.; Raitano S.; Kumar A.; Carvalho C.M.B.; Bagni C.; Ramocki M.B.; Araujo B.H.S.; Torres L.B.; Lupski J.R.; Van Esch H.; Muotri A.R.	2016	Altered neuronal network and rescue in a human MECP2 duplication model	Molecular Psychiatry	Yes	Not clear
139	Liu X, Campanac E, Cheung HH, Ziats MN, Canterel-Thouennon L, Raygada M, Baxendale V, Pang AL, Yang L, Swedo S, Thurm A, Lee TL, Fung KP, Chan WY, Hoffman DA, Rennert OM.	2017	Idiopathic Autism: Cellular and Molecular Phenotypes in Pluripotent Stem Cell-Derived Neurons	Mol Neurobiol	Yes	SCID
380	Deneault E, White SH, Rodrigues DC, Ross PJ, Faheem M, Zaslavsky K, Wang Z, Alexandrova R, Pellecchia G, Wei W, Piekna A, Kaur G, Howe JL, Kwan V, Thiruvahindrapuram B, Walker S, Lionel AC, Pasceri P, Merico D, Yuen RKC, Singh KK, Ellis J, Scherer SW.	2018	Complete Disruption of Autism-Susceptibility Genes by Gene Editing Predominantly Reduces Functional Connectivity of Isogenic Human Neurons	Stem Cell Reports	Yes	Not clear
212	Li S, Zhao H, Huang R, He L, Tian C, Huang H, Han X, Tang F, Lin Z, Deng S, Zhou J, Li Z.	2019	Generation of iPSC line (GIBHi001-A) from a patient with autism spectrum disorder	Stem Cell Res	Yes	NOD/SCID

continue...

...Continuation

Table 3S. Use of immunodeficient mice in teratoma formation assays to validate hiPSC pluripotency across reviewed studies

#	Author	Year	Title	Journal	Teratoma formation assays	Mouse strains
287	Song J, Yang X, Zhou Y, Chen L, Zhang X, Liu Z, Niu W, Zhan N, Fan X, Khan AA, Kuang Y, Song L, He G, Li W.	2019	Dysregulation of neuron differentiation in an autistic savant with exceptional memory	Mol Brain	Yes	SCID
168	Yang X, Liu Y, Zhou T, Zhang H, Dong R, Li Y, Liu N, Liu Y, Gai Z.	2019	An induced pluripotent stem cells line (SDQLCHi014-A) derived from urine cells of a patient with ASD and hyperactivity carrying a 303 kb de novo deletion at chr3p26.1 implicating GRM7 gene	Stem Cell Res	Yes	NOD/SCID
338	Yang X, Zhou T, Zhang H, Li Y, Dong R, Liu N, Pan G, Liu Y, Gai Z.	2019	Generation of an induced pluripotent stem cell line (SDQLCHi008-A) from a patient with ASD and DD carrying an 830 kb de novo deletion at chr7q11.22 including the exon 1 of AUTS2 gene	Stem Cell Res	Yes	Not clear
202	Zaslavsky K, Zhang WB, McCready FP, Rodrigues DC, Deneault E, Loo C, Zhao M, Ross PJ, El Hajjar J, Romm A, Thompson T, Piekna A, Wei W, Wang Z, Khattak S, Mufteev M, Pasceri P, Scherer SW, Salter MW, Ellis J.	2019	SHANK2 mutations associated with autism spectrum disorder cause hyperconnectivity of human neurons	Nat Neurosci	Yes	NOD/SCID
851	Kuebler B.; Aran B.; Flores R.; Pérez-Jurado L.A.; Veiga A.; Cuscó I.; Corominas R.	2020	Derivation of induced pluripotent stem cells (iPSCs) by retroviral transduction of skin fibroblasts from four patients suffering 7q11.23 microduplication syndrome	Stem Cell Research	Yes	SCID
277	Ross PJ, Zhang WB, Mok RSF, Zaslavsky K, Deneault E, D'Abate L, Rodrigues DC, Yuen RKC, Faheem M, Mufteev M, Piekna A, Wei W, Pasceri P, Landa RJ, Nagy A, Varga B, Salter MW, Scherer SW, Ellis J.	2020	Synaptic Dysfunction in Human Neurons With Autism-Associated Deletions in PTCHD1-AS	Biol Psychiatry	Yes	Not clear
264	Guo H, Jin ZL, Yao X, Park J, Gwon YP, Kim S, Kim KP, Cui XS, Kim NH, Yoo H, Han DW.	2021	Derivation of iPSC lines from two idiopathic ASD patients (OFi001-A, OFi002-A)	Stem Cell Res	Yes	NOD/SCID
265	Chen Z, Lv H, Yu J, Fang S, Li F.	2022	Generation of an induced pluripotent stem cell line SJTUXHi001-A from an autism spectrum disorder patient carrying a heterozygous mutation in HDAC8 (p.P359S)	Stem Cell Res	Yes	NOD/SCID
123	Ji Y, Lv H, Chen Z, Yu J, Fang S, Li F.	2022	Generation of a human induced pluripotent stem cell line (SJTUXHi002-A) from an individual with autism spectrum disorder carrying a heterozygous mutation in GRIA2	Stem Cell Res	Yes	SCID
334	Gong M, Li J, Qin Z, Machado Bressan Wilke MV, Liu Y, Li Q, Liu H, Liang C, Morales-Rosado JA, Cohen ASA, Hughes SS, Sullivan BR, Waddell V, van den Boogaard MH, van Jaarsveld RH, van Binsbergen E, van Gassen KL, Wang T, Hiatt SM, Amaral MD, Kelley WV, Zhao J, Feng W, Ren C, Yu Y, Boczek NJ, Ferber MJ, Lahner C, Elliott S, Ruan Y, Mignot C, Keren B, Xie H, Wang X, Popp B, Zweier C, Piard J, Coubes C, Mau-Them FT, Safraou H, Innes AM, Gauthier J, Michaud JL, Koboldt DC, Sylvie O, Willems M, Tan WH, Cogne B, Rieubland C, Braun D, McLean SD, Platzer K, Zacher P, Oppermann H, Evenepoel L, Blanc P, El Khattabi L, Haque N, Dsouza NR, Zimmermann MT, Urrutia R, Klee EW, Shen Y, Du H, Rappaport L, Liu CM, Chen X.	2024	MARK2 variants cause autism spectrum disorder via the downregulation of WNT/β-catenin signaling pathway	Am J Hum Genet	Yes	Not clear
